# Tumor-host signaling interaction reveals a systemic, age-dependent splenic immune influence on tumor development

**DOI:** 10.18632/oncotarget.6214

**Published:** 2015-10-21

**Authors:** Afshin Beheshti, Justin Wage, J. Tyson McDonald, Clare Lamont, Michael Peluso, Philip Hahnfeldt, Lynn Hlatky

**Affiliations:** ^1^ Division of Hematology/Oncology, Molecular Oncology Research Institute, Tufts Medical Center, Boston, MA, USA; ^2^ Center of Cancer Systems Biology, Tufts University School of Medicine, Boston, MA, USA; ^3^ Cancer Research Center, Hampton University, Hampton, VA, USA

**Keywords:** aging and cancer, tumor progression, tumor microenvironment, CD2, CD3e, Gerotarget

## Abstract

The concept of age-dependent host control of cancer development raises the natural question of how these effects manifest across the host tissue/organ types with which a tumor interacts, one important component of which is the aging immune system. To investigate this, changes in the spleen, an immune nexus in the mouse, was examined for its age-dependent interactive influence on the carcinogenesis process. The model is the C57BL/6 male mice (adolescent, young adult, middle-aged, and old or 68, 143, 551 and 736 days old respectively) with and without a syngeneic murine tumor implant. Through global transcriptome analysis, immune-related functions were found to be key regulators in the spleen associated with tumor progression as a function of age with CD2, CD3ε, CCL19, and CCL5 being the key molecules involved. Surprisingly, other than CCL5, all key factors and immune-related functions were not active in spleens from non-tumor bearing old mice. Our findings of age-dependent tumor-spleen signaling interaction suggest the existence of a global role of the aging host in carcinogenesis. Suggested is a new avenue for therapeutic improvement that capitalizes on the pervasive role of host aging in dictating the course of this disease.

## INTRODUCTION

Once considered a cell-based process, progression of existing cancers is now appreciated to involve elaborate tumor-host interactions, including, but not limited to, angiogenesis [1, 2], matrix remodeling [3, 4], and immune editing [5, 6]. Although the conduciveness of the host to tumor advancement is itself strongly dependent on host age, the direct role of age as a modifier of cancer progression remains unexplored. This is true despite age being among the strongest risk factors for cancer incidence. Epidemiological data show that from adolescence through middle age, cancer incidence increases with age, while during middle-age the incidence begins to decelerate and, for many tumor sites, it actually decreases at sufficiently advanced ages [7-9]. A global view of how age effects the host was recently discussed by López-Otin et al. [10] categorizing nine hallmarks of aging in the host, but little was mentioned on the direct impact of these hallmarks on cancer progression other than acknowledging that aging and cancer can be considered as two entities manifesting many of the same overall processes (i.e. increases in mutations and cellular damage). Most of the molecular factors involved in the nine hallmarks of aging stem from individual organs. The impact of individual organs is usually not considered unless discussing the direct tumor environment (in other words the tumor microenvironment) or the organs which are susceptible to metastases for a particular cancer [11]. The influence of organs, individually or as a whole system, is vastly underappreciated and not well understood for cancer risk [12]. Certain organs, such as the spleen, controls hematopoiesis which has the ability to modulate cancer risk for the host [13]. Changes as a function of age with the immune system stemming from both the spleen and liver also have potential to effect cancer risk and tumor progression [14]. For the most part there is limited research on how the effects of individual organs and the interplay between organs will impact cancer risk and more importantly how age perturbs the whole system. The impact of understanding how the effects of organs change as a function of age and how this will impact cancer risk can improve current therapeutics and create age dependent therapy for cancer.

Since observing changes due to tumor burden as function of age for an entire organismal system is beyond the scope of one paper, we first will focus on splenic modulations that occur with or without tumor burden as a function of age. The spleen is the largest organ with similar functions to lymph nodes and acts as a blood filter which plays a key role in initiating immune response and reaction to pathogens, viruses, and other stress caused by diseases such as cancer [13, 15]. The spleen is composed of two functionally different compartments: 1. the red pulp, which is acts as a blood filter that removes foreign material and damaged or old erythrocytes; and 2. the white pulp, which is composed of three compartments (periarteriolar lymphoid sheath (PALS), the follicles, and the marginal zone) and is responsible for initiating immune responses to blood-borne antigens [13, 16-18]. It has been reported that the spleen weight as a function of age in healthy individuals in both rodents and humans remain fairly constant after adolescence [13, 17, 19]. Also in healthy humans, there has been no age related differences observed with internal morphology of the spleen, i.e. differences in the number of PALS, follicles, or B and T cell density [16, 17]. In general mouse and human spleens are mostly similar in anatomy with human spleens displaying a more organized marginal zone [13, 15]. Significant splenic weight changes occur from outside influences causing the spleen to be either be congested and contracted [17]. Cancer associated splenomegaly (or enlarged spleen) has been shown to be caused by either metastasizes to the spleen or blood based cancers (i.e. lymphomas, leukemia, etc.) [15]. In mice, splenomegaly also has been attributed tumor cell transplantable models, in addition to a vast array of diseases [15]. Limited research has shown that the impact of distant tumors to the spleen can impact splenic function. For example newly formed monocytic cells produced by the spleen can migrate to the tumor microenvironment and create new tumor-associated macrophages during tumor progression [15, 20]. In general it has been shown that tumor bearing mice have an increase in monocytic cells in the spleen [21]. Signals originated from the tumor have also been shown to provide antigen tolerance through the spleen that slows tumor progression from murine studies involving splenectomy studies with colon carcinoma, mammary carcinoma, and melanoma [15, 22, 23]. The limited research on the effects of long distance tumor burden starts to show the importance of considering the organism as a whole a system.

Rather than focusing on certain factors, we approached this subject by studying the host (i.e. spleen) as a function of age as a whole system with and without tumor burden. We previously showed the effects of tumor progression as a function of age, where it was revealed that old hosts provide an environment for the retarded tumor progression with TGFβ1 being the key player involved within the tumor [9]. Here we expand on these previous studies by comparing the spleens from the same tumor bearing mice with spleens from non-tumor bearing mice as a function of age. Global transcriptome studies on the spleen with and without the impact of the tumor surprisingly revealed up-regulated immune related functions in old hosts with CD2 [24], CD3ε [25], CCL5 [26], and CCL19 [15, 18] being key T-cell related factors. This analysis provides a preliminary mechanistic understanding of several host factors with a robust effect on tumor progression with age that may also lead to novel therapeutic targets.

## RESULTS

### Morphological changes occurring in the spleen as a function of age with and without tumor burden

**Figure 1:**
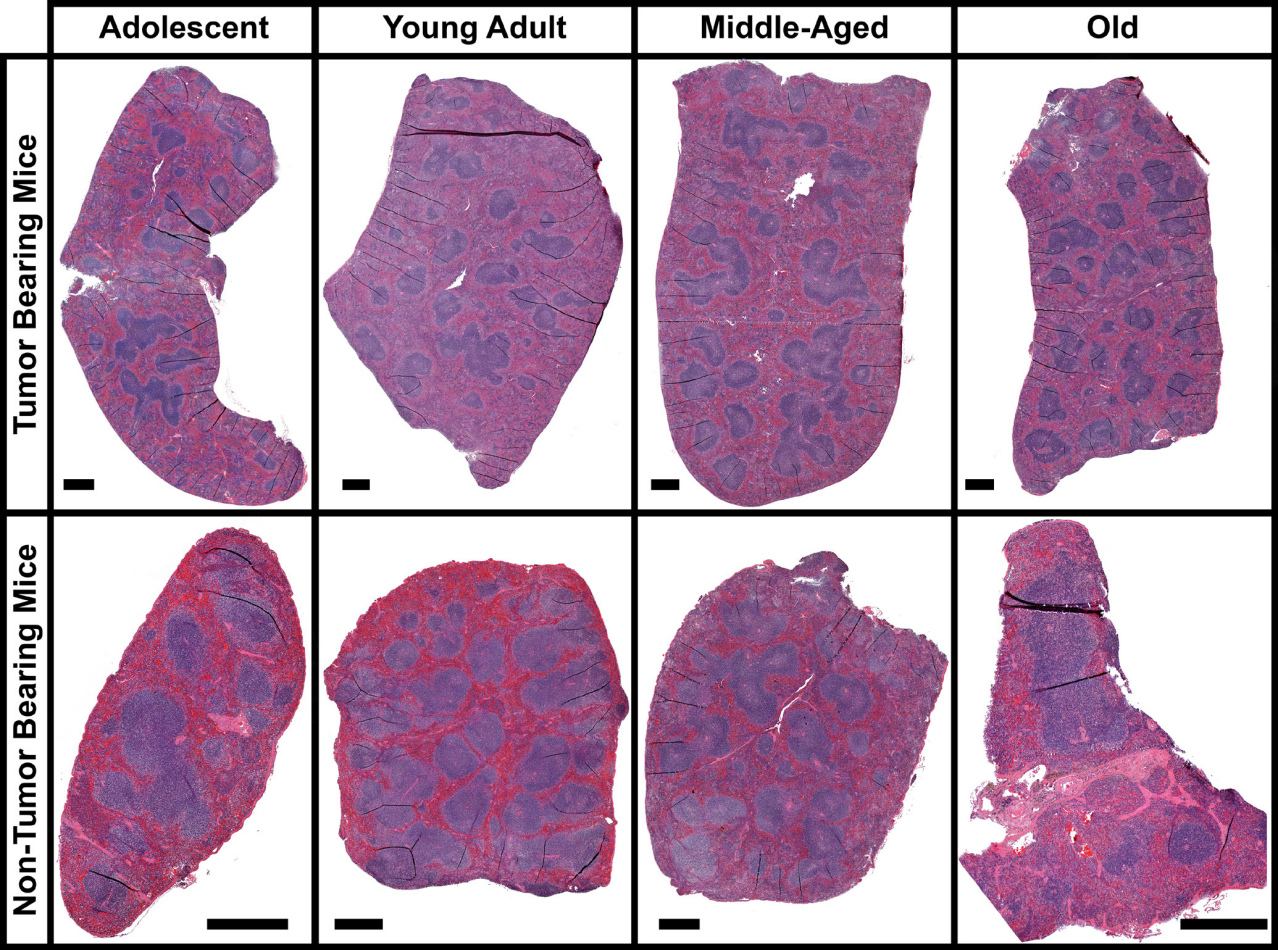
Morphology of the spleen for tumor and non-tumor bearing mice as a function of age Images of entire spleen sections stained with Hematoxylin and Eosin (H&E). The black scale bar represents 500μm.

**Figure 2:**
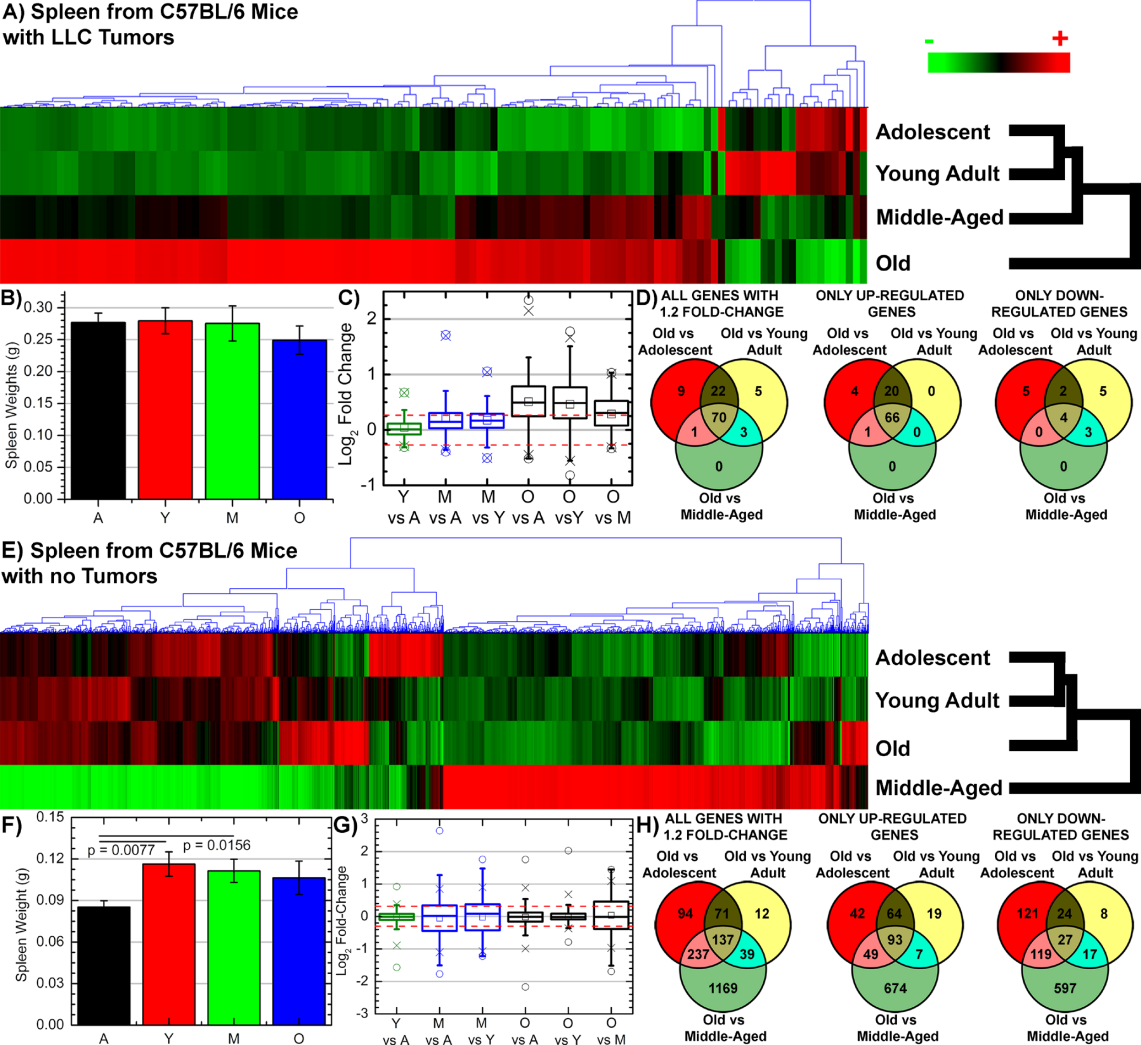
Gene regulation and morphology changes for spleens as a function of host age from tumor and non-tumor bearing C57BL/6 mice **A**. & **E**. Hierarchical clustering of genes by average linkage (UPGMA) and Euclidean distance calculation between age groups adolescent, young adult, middle-aged, and old of the 122 significant genes for spleen tissue from tumor bearing mice and 2102 significant genes for spleen tissue non-tumor bearing mice with one-way ANOVA, FDR < 0.05. **B**. & **F**. Weights of spleens from C57BL/6 mice with different age groups from tumor bearing and non-tumor bearing mice: adolescent (A), young adult (Y), middle-aged (M), and old (O). **C**. & **G**. Average signal log_2_ fold-change comparing adolescent (A), young adult (Y), middle-aged (M), and old (O) to each other. Whiskers show the range of the outliers, with max and min values as O and the 1 and 99^th^ percentile outliers as X. **D**. & **H**. Venn diagrams of the genes with 1.2 fold change for comparisons between old spleen samples to all other samples with separate Venn diagrams for the only the up- and down-regulated genes.

There was little change for internal differences in the spleen as a function of age. H&E stain reveals the overall internal structural appearance of spleens at different ages with and without tumor burden (Fig. 1). For both tumor and non-tumor bearing mice there are no significant changes occurring with the red and white pulp areas including the marginal zones, follicles, or PALS as a function of age. In general it has been reported that as a function of age spleen weights and morphological changes stay fairly constant [16, 17]. Morphological changes occur in the spleen when tumor burden is applied to the overall host, but in general no significant differences are noticeable as a function of age. The most apparent differences occur with spleen weight when comparing tumor bearing mice to non-tumor bearing mice (Fig. 2B). On average, splenic weight doubles in size for tumor bearing mice independent of age. In mice splenomegaly has been reported to occur with many tumor implant models [15, 17]. As a function of age there are no significant changes other than for adolescent non-tumor bearing mice which have an overall reduction in spleen size compared to all other age groups.

### Tumor burden creates unique molecular profile in the spleen as a function of age

Global transcriptome analysis revealed distinct difference between tumor bearing mice compared to non-tumor bearing mice as a function of age. For both tumor and non-tumor bearing mice young and adolescent hosts are the most genetically similar. This pattern is also reflected from observing 1.2 fold-change (log_2_) differences between each age group (Figs. 2C and 2G). For both tumor and non-tumor bearing hosts, spleens from young and adolescent hosts show minimal significant fold-change differences. In the oldest age groups, tumor bearing hosts show mostly up-regulation compared to all age groups (Figs. 2C and 2D), while in contrast spleens from non-tumor bearing host show most genes below the 1.2 fold-change cutoff with an equal distribution of genes being up- and down-regulated (Figs 2G and 2F). Surprisingly, global transcriptome analysis show a distinct difference in molecular profile of the spleen as a function age when a tumor is added the overall host system. Overall clustering of the significant genes from spleens of tumor bearing mice (122 genes with a FDR < 0.05) demonstrated that the oldest mice have the most significantly different profile compared to all other age groups with increasing similarity as a function of decreasing age (Fig. 2A). While for non-tumor bearing hosts, the significant list of genes (2102 genes with a FDR < 0.05) revealed that spleens from middle-aged mice have the most significantly different profile followed by old mice (Fig. 2E). This data suggests the molecular response in the spleen due to the systemic pressure of tumor burden is a function of host age.

### Immune system regulation for spleens in tumor bearing mice

**Figure 3:**
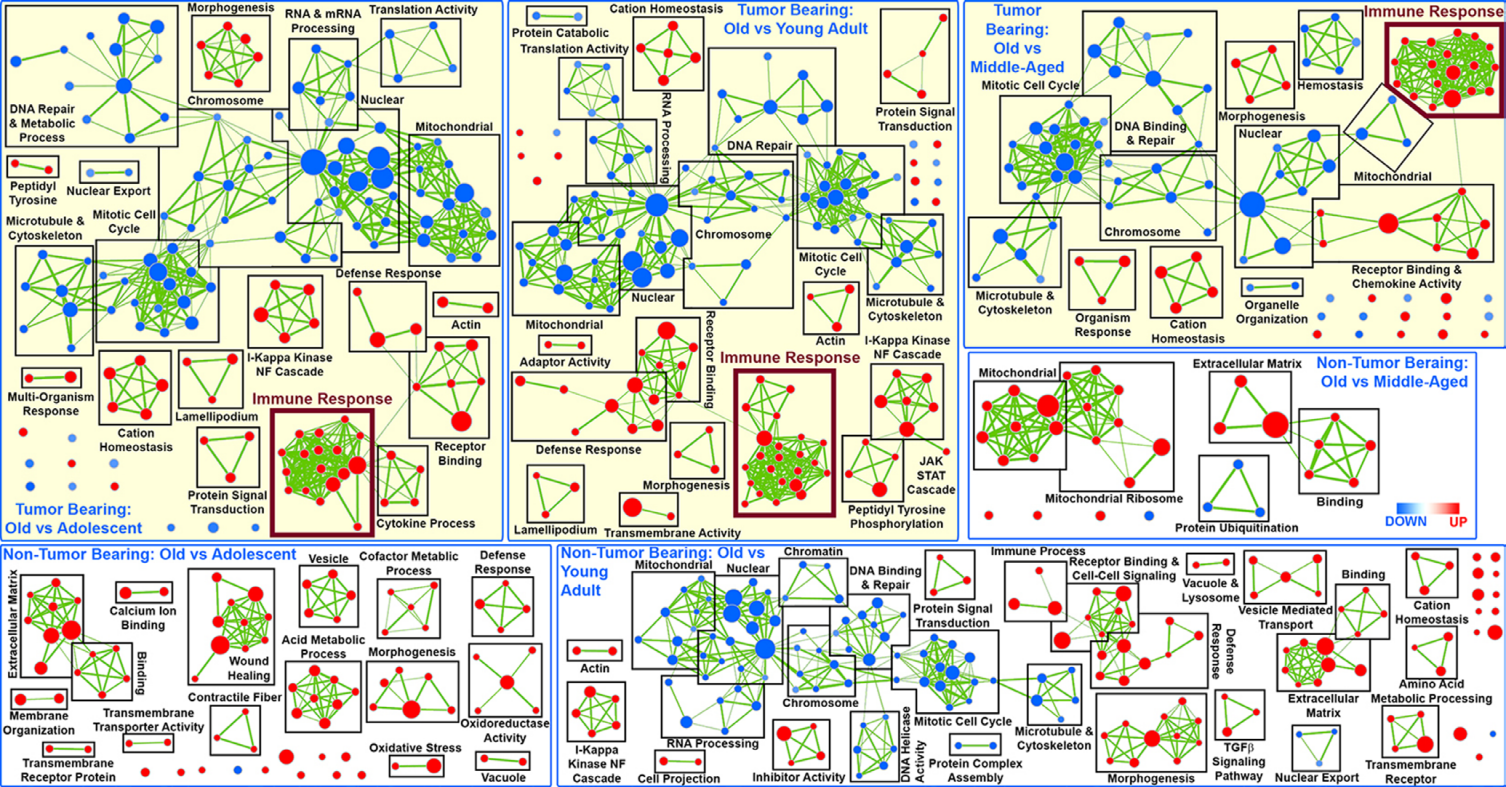
Network representation of Gene Set Enrichment Analysis (GSEA) for GO C5 gene sets in the spleen only for old hosts compared to all other age groups (Adolescent, Young Adult, and Middle-Aged) from tumor bearing and non-tumor bearing mice Leading edge analysis with a FDR < 0.05 determined significant gene sets enriched for each group. The size of each node reflects the amount of molecules involved for each gene set. The edge (green lines) represents the number of genes associated with the overlap of two gene sets (or nodes) that the edge connects. Clusters were named according to common function in each grouping. Upregulated gene sets were denoted with red color and downregulated gene sets were denoted by blue color. Common up-regulated clusters related to immune response is indicated by larger font and thicker box in maroon.

More in depth analysis of the transcriptome data continued to reveal focused functions and factors for tumor bearing mice as a function of age. Gene Set Enrichment Analysis (GSEA) reveals several distinct functions for old hosts compared to all other age groups for tumor bearing mice, which non-tumor bearing mice do not exhibit (Fig. 3. and Supplemental Tables 1 and 2). In general, for non-tumor bearing mice, old compared to adolescent mice significantly differ only by up-regulation of gene sets (or functions), for instance, related to extracellular matrix and binding. Depending on the age comparison for spleens in non-tumor bearing mice, different functions are up- or down-regulated without a common theme as a function of age (Fig. 3 and Supplemental Table 2). The presence of a tumor seems to provide some stability to this naturally occurring biological noise as a function of age and induces common functions in the spleen that are affected in old hosts compared to all other age groups (Fig. 3 and Supplemental Table 1). There are several gene sets that are commonly down-regulated for old hosts when compared to all other age groups, which are related to mitotic cell cycle, DNA repair functions, nuclear related functions, mitochondrial functions, RNA processing, chromosome changes, and cytoskeletal related functions. The down regulation of these functions are in agreement with what has been shown in the literature to be associated with age related factors in older hosts [10, 27, 28].

Surprisingly, only for tumor bearing mice immune related factors are commonly being up-regulated with increasing age (Fig. 3 and Supplemental Table 1). With GSEA analysis, in spleens of old tumor bearing mice compared to all age other groups we observe a large cluster of up-regulated immune related functions ranging from T-cell, leukocyte, and lymphocyte activation to more general immune functions (i.e. adaptive immune system regulation) (Supplemental Table 1). For spleens from old non-tumor mice, there is some minimal activity of up-regulation of immune related functions, but this does not occur for all age comparisons to old hosts and is only represented by very few gene sets when it does appear. Again presence of a tumor in the overall host seems to reduce biological noise and produce a unifying and focused behavior in spleens of old hosts to activate immune related functions to combat the presence of the tumor.

Independent biofunction predictions made by Ingenuity Pathway Analysis (IPA) also confirm that there is an increase in immune related functions for spleens from old tumor bearing mice. As before, for non-tumor bearing mice no distinct pattern of functions occur in the spleen for old mice compared to any of the age groups (Supplemental Table 4). Presence of a tumor provides a unifying theme for spleens from older hosts with a predicted increase in immune related factors through IPA biofunction analysis. More specifically there is a decrease in apoptosis and cell death with an increase in cell movement, migration, and adhesion of splenic immune cells for old hosts when compared to younger hosts (Supplemental Table 3).

**Figure 4:**
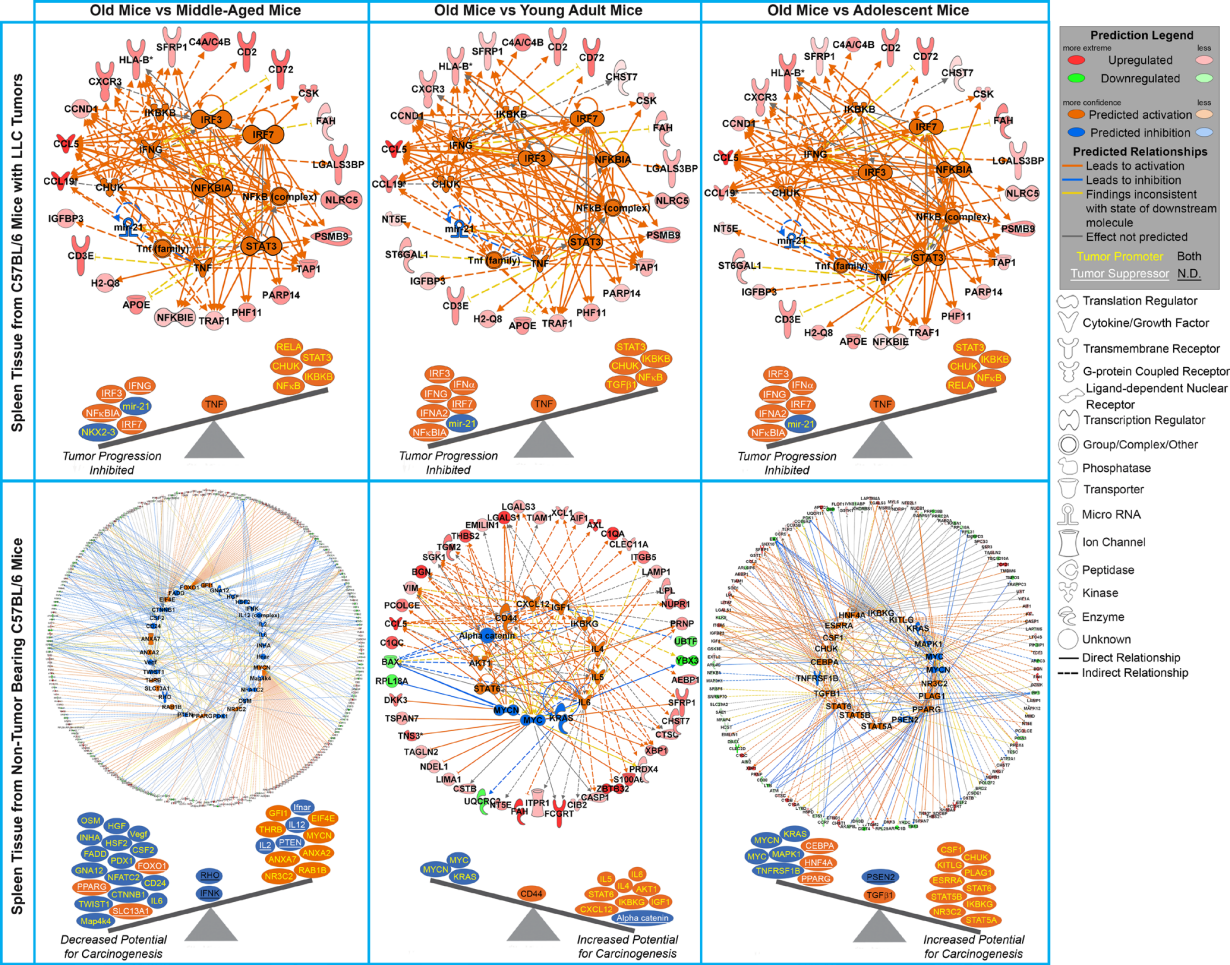
Common Upstream regulators determined by Ingenuity Pathway Analysis (IPA) software from the significant genes for old mice compared to all other age groups for tumor bearing and tumor non-bearing mice Gene network depiction of the common upstream regulators for old mice compared to all other age groups predicted to be either activated (orange) or inhibited (blue) determined by IPA software. Specific upregulated (red) and downregulated (green) genes from the experimental data set involved in determining the activation state of the upstream regulator are shown with direct (solid lines) and indirect (dashed lines) relationships to the upstream regulators. A predicted relationship is color coded to indicate whether it leads to activation (orange) or inhibition (blue). Relationships that are inconsistent with the prediction (yellow) or have an undetermined effect (grey) are also shown. The darker the shade of green or red, the greater the fold change. Below each network is a schematic of the activation states of the upstream regulators from Table 1 illustrating the balance between the tumor promoters (text in yellow) and tumor suppressors (text in white and underlined) with a predicted activation (circled in orange) or predicted inhibition (circled in blue). Upstream regulators that are predicted to have both tumor promoting and suppressing characteristics are shown with black text.

Additionally, there are specific immune related regulators being predicted to be involved in spleens of tumor bearing old mice which are not observed in non-tumor bearing mice through IPA upstream regulator analysis. For spleens from tumor bearing old mice majority of the upstream regulators are either interferons (*IFNγ, IFNα, IRF3, IRF7,* and *IFNA2*) or are related to the immune system (*IKBKB, CHUK, NFκB, NFκBIA, STAT3*, and *mir-21*) (Fig. 4 and Supplemental Table 5) and are predicted to be up-regulated. Interferons provide communication between cells to allow the immune system to become active and allow a defense against pathogens or tumors [29-31]. Other factors, such as *STAT3* [32], cooperate with the immune system to active immune related functions in the host to reduce tumor progression. Spleens from non-tumor bearing mice did not have common factors and patterns for old mice compared to all age groups. There were a few upstream regulators commonly regulated for middle-aged mice compared to all other age groups without a very unifying theme (Fig. 4 and Supplemental Table 6). Overall transcriptome data for spleens from tumor bearing old mice demonstrate an activation and increase of immune related factors when compared to all younger age groups. This surprising trend is only observed in tumor-bearing mice, with non-tumor bearing mice having no distinct pattern or commonality within age groups and providing what we are referring to as biological noise.

### Molecular factors from the spleen can predict the tumor dynamics

Factors from the spleen provide an immense impact on how the entire system or host will react to outside influences (i.e. cancer) and react to the outcome of such insults. It is observed that presence of a tumor will influence factors of the spleen to affect progression as a function of age. Upstream regulator analysis using Ingenuity Pathway Analysis (IPA) in conjunction with information on reported impact on tumor progression from the literature (Fig. 4 and Supplemental Tables 5 and 6) provide evidence for age-dependent impact of the spleen on tumor progression. Suggested is a stable shift of upstream regulators toward inhibition of tumor progression with increasing age for tumor bearing mice (Fig. 4). This predicted tumor inhibition is in agreement with the tumor dynamics previously showing that old mice had a slower progression compared to all other age groups [9]. For non-tumor bearing mice, the upstream regulator analysis showed an influence on the potential for carcinogenesis in lack of a tumor presence. Interestingly, for non-tumor bearing mice only old mice compared to middle-aged mice show decreased potential for carcinogenesis. For all other age groups old mice exhibit an increased potential for carcinogenesis (Fig. 4). This indicates that factors in old hosts, particularly immune related, which have potential to increase tumor formation make a phenotypic switch, once a tumor is present, providing host-age related resistance as a means to decrease tumor progression.

### Key immune related factors from the spleen influencing tumor progression in old hosts

**Figure 5:**
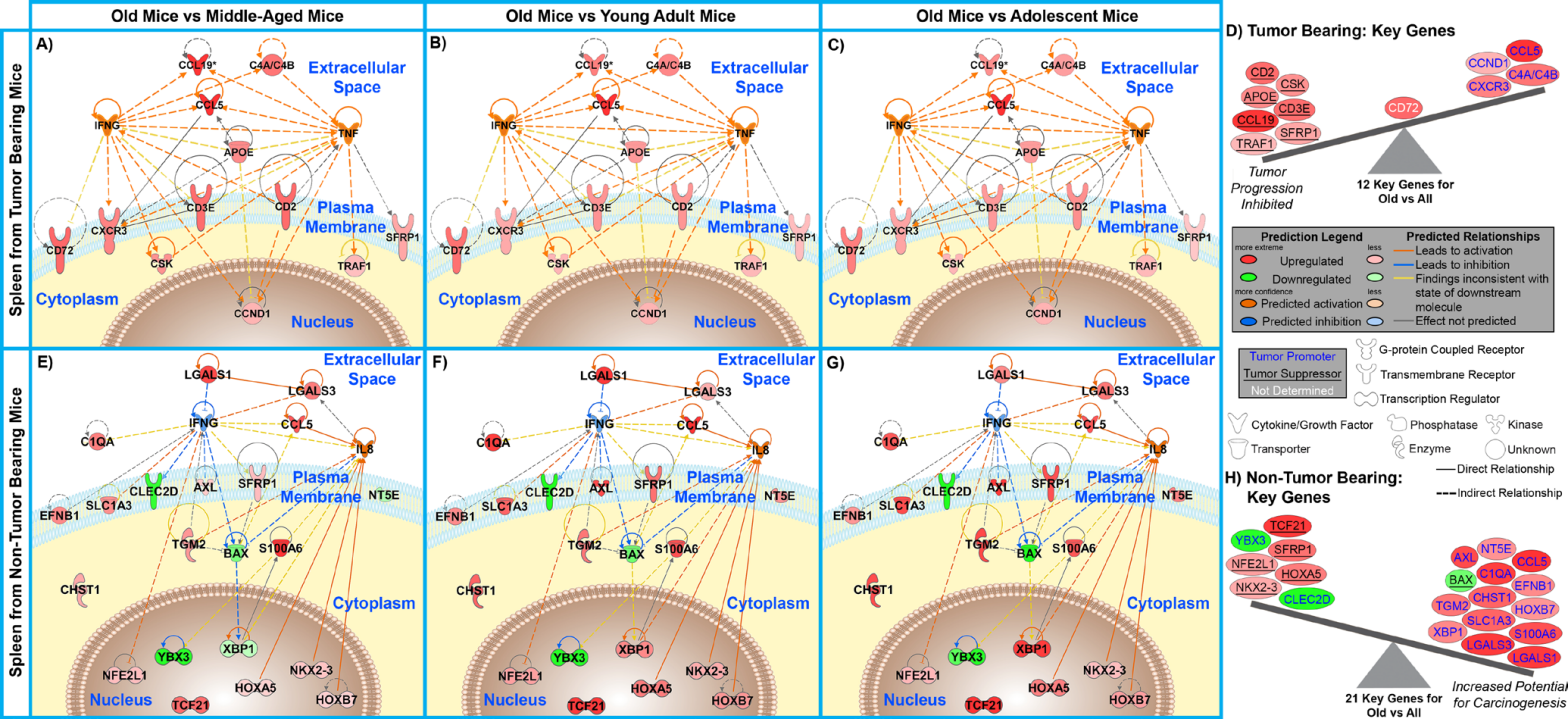
Significant molecular factors involved in the spleen for tumor bearing and non-tumor bearing old hosts **A**. – **C**. & **E**. – **G**. Gene network analysis for key genes involved in age-dependent splenic changes for old mice compared to all other age groups. Pathway analysis was done with Ingenuity Pathway Analysis (IPA) software. IFNG, IL5, and TNF were added to the network to provide relations and connections between all genes. Predicted activity due to other genes is also shown for these two genes. Log_2_ fold changes to the gene expression were used to obtain different shades of green for regulation levels for 1.2 fold change in down-regulated genes, while different shades of red depict regulation levels for 1.2 fold change in up-regulated genes. The darker the shade of green or red, the greater the fold change. **D**. & **H**. A schematic of the significant genes (Table 4), determined to be key in regulating many functions at older ages, illustrating the balance between the tumor promoters (text in yellow) and tumor suppressors (text in white and underlined) with the Log_2_ fold change color coded as before. Genes that have no determined effect on tumor progression appear in white text.

An unbiased analysis was used to determine key factors from the spleen involved with tumor and non-tumor bearing hosts. Key genes driving the observed age-dependent modulation in the spleen were determined by comparing common genes involved in the predicted upstream regulator analysis to the biofunction analysis for old spleen samples compared to all age groups for both tumor and non-tumor bearing mice. This resulted in 12 and 21 key genes for spleens from tumor and non-tumor bearing old mice compared to all age groups (Fig. 5 and Supplemental Tables 7 and 8). When relating these key genes to the reported impact of tumor progression in the literature, once again for tumor bearing old mice the key genes in the spleen show an inhibition of tumor progression while for non-tumor bearing old mice demonstrate an increased potential for carcinogenesis.

To further reduce the focus to the essential genes involved with either the tumor or non-tumor bearing mice, the overlap of genes from the above analysis was compared to the genes involved with GSEA analysis (Fig. 3 and Supplemental Tables 1 and 2). For both tumor and non-tumor bearing mice this lead to four key genes for each condition of old mice compared to all other age groups. For non-tumor bearing old mice the four genes are *C1QA* [33, 34], *LGALS3* [35], *SFRP1* [36], and *CCL5* [26]. C1QA is a subcomponent of C1Q which is a major part of the complement system and is involved with endothelial cells to help promote angiogenesis, remove infectious agent, remove apoptotic cells, and interact with immune complexes [33]. Also C1Q production has been shown to be bound to the germinal centers in spleens [34]. LGALS3 has an essential role in the innate and adaptive immune system, through interacting with T regulatory cells [35]. SFRP1 is involved in regulating apoptosis through Wnt signaling pathway and down-regulation or loss of SFRP1 has been shown to provide poor prognosis for cancer patients [36]. CCL5 is a chemotactic cytokine for T cells and is involved with recruitment of leukocytes to inflammatory sites [26]. These four key genes for non-tumor bearing old mice were also all up-regulated when compared to all the younger age groups. The overall balance of these four genes with regards to tumor influence indicates that there is a potential of increased carcinogenesis for old mice when compared to all younger age groups.

For tumor bearing mice the four key remaining genes are all up-regulated and are *CD2* [24], *CD3ε* [25], *CCL19* [15, 18], and *CCL5*. One key gene, CCL5, behaves independently of tumor burden and is up-regulated as a function of old age in the spleen. The other three genes are only up-regulated in old mice once tumor burden is present. CD2 is a calcium-independent cell adhesion glycoprotein which is expressed on T cells, natural killer (NK) cells, and thymocytes. It has been shown that it plays a subtle role in T cell activation whereby CD2 deficient mice require higher concentration of antigen in order to initiate a T cell response via production of interleukin 2 (IL-2) in the cytoplasm after interaction with its target molecule [24]. CD3ε is a component of the T-cell receptor complex which plays an essential role in T cell survival. Deficiencies of CD3ε have been shown to occur in immunodeficient patients and also in many cancers [25]. CCL19 is a cytokine that is involved in attracting T cells and dendritic cells to the T cell region of the white pulp in spleens [18]. CCL19 has also been shown to occur in low expression in spleens of hosts without the presence of a tumor [37]. These three key genes, up-regulated only in tumor bearing old mice, all are commonly involved with T cell activation and recruitment and collectively play an essential role in the immune systems involvement to reduce tumor progression.

**Figure 6:**
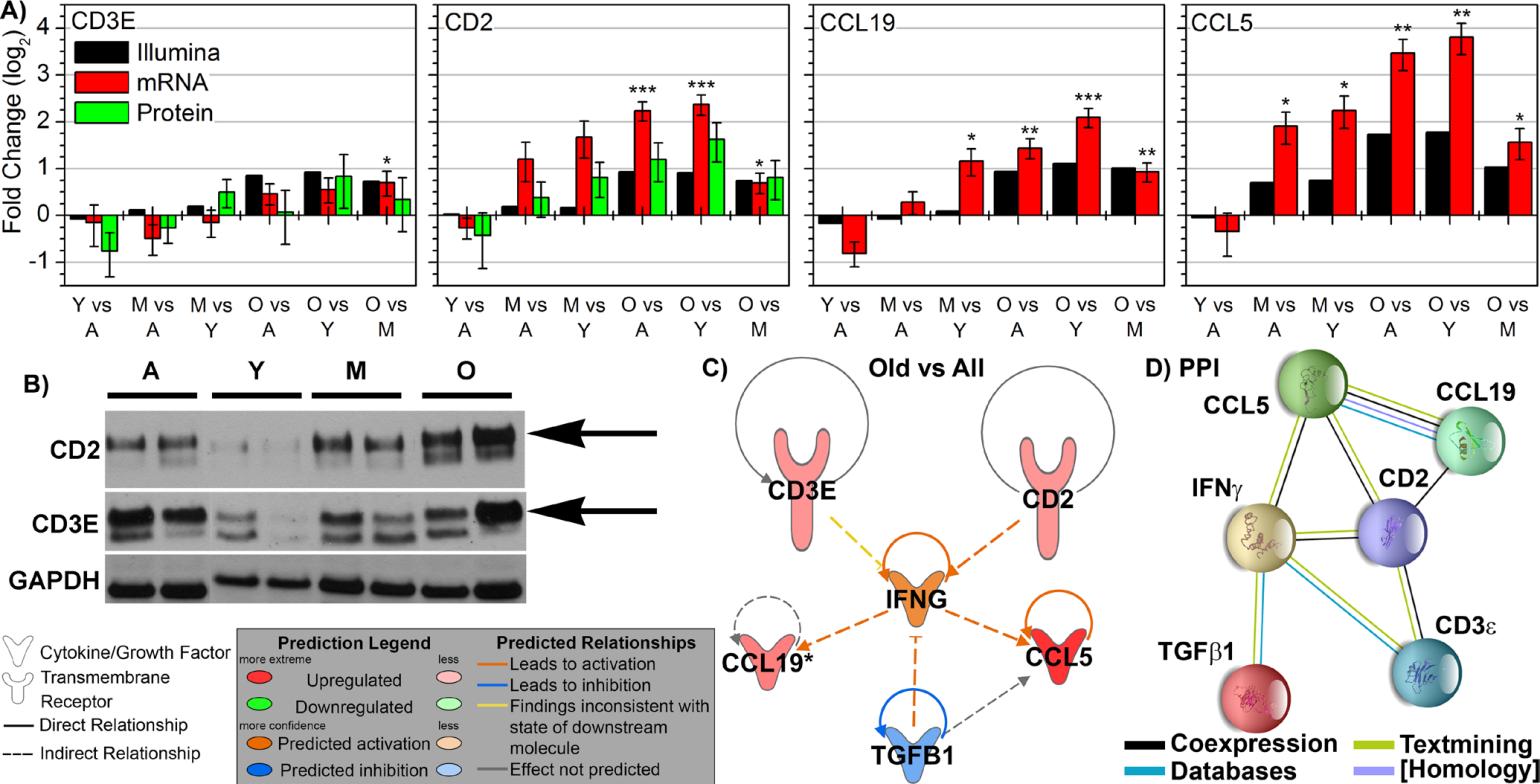
Key immune molecular factors in spleen related to key molecule affecting tumor progression as a function of age **A**. The mRNA expression determined by real-Time PCR (RTPCR) for CCL19, CD2, CCL5, and CD3E and quantified protein expression from western blots for CD2 and CD3E compared to the gene expression observed by Illumina. Log_2_ fold changes for mRNA and protein expression were determined for each group. * p < 0.05. **B**. Representative western blots for CD2 and CD3e with technical control GAPDH for Adolescent (A), Young Adult (Y), Middle-Aged (M), and Old (O) mice **C**. Pathway analysis was done with Ingenuity Pathway Analysis (IPA) software. INFG was added to the network to provide relations and connections between all genes to TGFβ1. Predicted activity due to other genes is also shown for INFG. **D**. A Protein-Protein Interaction (PPI) network was constructed using STRING for the key genes including the relation to TGFβ1 through IFNG. The nodes are connected through lines indicating the relationship between each protein. The color of the lines indicates the evidence of the connection between each protein. The protein structure is shown within the node.

Confirmation of the four key genes in the spleen associated with tumor bearing old mice was performed by real-time PCR (RT-PCR) and westerns. The mRNA levels were confirmed through RT-PCR and were shown to match the gene expression values closely (Fig. 6A). Also for CD2 and CD3ε western blots were performed and were shown through quantification of the specific bands that the protein levels match the same pattern and trends observed with mRNA levels (Figs. 6A and 6B). Since low expression and amount of CCL19 has been reported in the spleen [37], western blots do not provide enough sensitivity to detect CCL19 in the spleen. Since CCL5 was discovered to be up-regulated in the spleen of old mice independent of tumor burden, we don't consider it as important as the other factors for old tumor bearing mice.

Surprisingly, the four key factors in the spleen of tumor bearing old mice are related to the key factor determined to be in involved in the tumor. Previously we have shown that TGFβ1 was the key player involved in tumors of old hosts involved in the inhibition of tumor progression [9]. A network was generated using IPA from transcriptome data predicting the relationship to *TGFβ1* (Fig. 6C). TGFβ1 was predicted to be inhibited in this network including the four key factors and *IFNγ* (which was also predicted it be activated from upstream regulator analysis (Fig. 4)). This prediction amazingly and independently agrees with transcriptome and protein results obtained from the tumor [9]. A protein-protein interaction (PPI) network for the same factors was also constructed through STRING [38] (Fig. 6D). Since transcriptome interactions and protein interactions not always coincide, it is important to observe both interactions. The PPI network demonstrated a direct relationship to TGFβ1 through IFNγ (Fig. 6D). For non-tumor bearing old mice the four key factors did not have a direct network connection or a unifying network similar to tumor bearing old mice (data not shown). Through the analysis from the spleen there is surprising evidence of how a system as a whole (the system being the whole organism) can provide focused long distance effects on a tumor.

## DISCUSSION

Limitations currently occur with the understanding of age related cancer risks which impede progress on assessment on both early detection and therapies. Current understanding on age related cancer risk is mainly based on observational studies mostly from clinical studies and cases [7, 39]. There is a lack of detailed temporally-oriented molecular studies to correctly assess not only the perturbations that occur with age affecting cancer risk, but the consequences of these perturbations on the continuing course of cancer progression leading up to disease presentation. Gaining predictive insight with a risk model of how age changes the host as a whole can inform the problem of ameliorating that risk. Quantifying how the specific molecular factors from various organs (e.g. spleen) change with age with and without tumor burden have potential to provide new biomarkers for both early detection of cancer and future advancement with individualized therapeutic options.

Here we present the effects of a tumor-host interaction driven by splenic changes as a function of age. The spleen functions as the body's largest filter for the blood and is heavily involved with both innate and adaptive immune system response to pathogens both locally and for the whole host or system [13, 18, 40]. Minimal morphological splenic changes have been reported to change as a function of age [13], in agreement with our findings (Fig. 1). On a cellular level it has been reported that as a function of age a decrease of up to 80% has been observed in lymphocyte counts, with a corresponding increase in reticular cells and macrophages in the white pulp [13]. It has also been noted in rodents that fewer germinal centers can occur with increasing age [13], but that is not observed with the present data.

Important changes occurring as a function of age are not apparent on the morphological level, but on the cellular and molecular level in the spleen when comparing mice with and without tumor burden. Global transcriptome data revealed distinct differences occurring for older mice with tumor burden (Fig. 2). Surprisingly, tumor burden reduces the natural biological noise that occurs for molecular factors as a function of age and provides a singular factor that arises above the noise for old hosts to reduce tumor progression. We observed that tumor burden impacts spleens in older hosts by unexpectedly activating and up-regulating immune related factors when compared to all younger age groups (Fig. 3 and Supplemental Tables 1, 3, and 5) while for non-tumor bearing hosts no apparent common function occurs as a function of age in the spleen. Other more expected functions are shown to be commonly reduced for old tumor bearing hosts, such as nuclear functions, cell cycle, mitochondrial functions, and cytoskeletal related functions. Again for non-tumor bearing hosts there are no apparent common functions occurring between different age groups which we account to natural biological noise that occur as a function of age. Recently Lopez-Otin et al., identified nine hallmarks of aging with one hallmark being alterations intercellular communication which can be manifested as a pro-inflammatory phenotype [10]. They refer to this continuous low-grade increase in inflammation with increasing age as inflammaging which was first described by Franceschi et al [41]. It is believed that inflammaging is a consequence of reduced responsiveness and decline of communication between all immune cells as a function of age which is referred to as immunosenescence [41, 42]. It is commonly accepted that immunosenescence is a prominent feature associated with old age that impacts both the innate and adaptive immune system of the host, which in addition to inflammaging, results in decrease in diminished self-renewal capacity of hematopoietic stem cells, decline in total number of phagocytes and NK cells, reduced humoral immunity, and overall reduced functional capacity of both B and T cells [42, 43].

The aged mice used in this study would be expected to have a decrease in immune functions that would result in an increase in tumor progression compared to younger ages. For non-tumor bearing old mice, this overall theme seems to coincide with the results obtained. Old mice compared to younger age groups exhibited an overall increased potential for carcinogenesis based on predicted upstream regulators and determined key genes (Figs. 4 and 5). Predicted factors determined from transcriptome data also show indication of immunosenescence and inflammaging occurring in the spleen for old hosts including: increased apoptosis of B lymphocytes, reduced quantity of both leukocytes and lymphocytes, increased phagocytosis related to increased chronic inflammation, and increased production of reactive oxygen species (which is again related to an increase chronic inflammation [44]) (Supplemental Tables 2 and 4).

However, once a tumor is introduced to the system or organism no indication of immunosuppression or inflammaging occurs and a common theme of increased immune system regulation exist for old hosts (Fig. 3 and Supplemental Tables 1, 3, and 5). Predictions based on upstream regulator analysis and key genes also indicate that an overall inhibition should occur for tumor progression (Figs. 4 and 5), which agrees with the actual reduced tumor growth that was discussed in an earlier publication [9]. When observing specific factors involved in spleens for old tumor bearing mice, there is indication that the spleen protects the host from carcinogenesis and may maintain cellular homeostasis through a process referred to as immunosurveillance [44]. This process has been associated with reducing immunosuppression and chronic inflammation related to inflammaging [14, 43, 44]. Through upstream regulator analysis it was predicted that several interferons, IFNγ, IFNα, IRF3, IRF7, and IFNA2, were up-regulated for spleens from old tumor bearing mice. Activation of these interferons have been associated with tumor suppression by interacting with the immune system [30, 31, 45, 46]. More specifically IFNγ has been shown to directly activate immune cells (i.e. macrophages and NK cells) to reduce tumor growth [44]. Although IFNγ was not predicted to be activated or inhibited with upstream regulator analysis for old non-tumor bearing mice, we observed that the overall influence of the key genes involved for this condition down-regulated IFNγ (Fig. 5).

Further analysis revealed that four key molecules impacting the increase in immune activity in the spleen for old tumor bearing mice provided the mechanistic link to factors involved directly in the tumor. It was revealed that the following immune related factors were key factors being commonly up-regulated for old tumor bearing hosts compared to all younger age groups: CD2, CD3ε (or CD3E), CCL19, and CCL5 (Fig. 6). Only CCL5 overlapped as a key factor for non-tumor bearing old mice, which means that CCL5 can be considered a natural factor that will be up-regulated as a function of age in the spleen regardless of tumor burden. CCL5 (also known as RANTES) is a key pro-inflammatory chemokine involved in migration and recruitment of T cells, dendritic cells, eosinophils, NK cells, mast cells, and basophils [26]. Since impact of this molecule in the spleen is observed as a function of age independent of tumor burden, more focus will be spent on the three factors which are also being up-regulated. All key molecules in the spleen of tumor bearing old hosts (CD2, CD3ε, CCL19, and CCL5) (also been reported to exist in the spleen) leads to immune cell survival and function through increased T-cell survival and activity [18, 24-26]. CD2 expression has been associated with activation and initiation of T cells and in immune response from both immune and non-immune associated tissue [24, 47]. Down-regulation of CD3ε hinders immunosurveillance and has been associated with T cell apoptosis which helps facilitate the role of tumor immune evasion [25]. It has been reported that CCL19 promotes immune response through co-stimulating T cell activation and increasing T cell interactions with antigen-presenting cells (APCs), dendritic cells, and B cells allowing for lymphocyte migration and homing [40].

Combination of these four key factors creates an increased environment for immune response and immunosurveillance that may reduce and inhibit tumor progression. As stated earlier for aged hosts without tumor burden, we observe a decrease in functions related to lymphocytes and leukocytes and the four key factors (C1QA [33, 34], LGALS3 [35], SFRP1 [36], and CCL5 [26]) that do not provide a collective activation of the immune system needed to suppress tumor growth as is commonly accepted in aged hosts. This surprising increase of T cell activity from spleens of older hosts due to CD2, CD3ε, CCL19, and CCL5 directly has impacted the decrease in tumor growth we observed in our earlier publication [9]. We demonstrated directly from tumor tissue (which contains both tumor and stromal components) that TGFβ1 was decreased in tumors of older hosts. For late stage tumors, TGFβ1 is known to be tumor promoter and help facilitate more aggressive tumors [11]. Amazingly through IFN a simple network relationship for both the transcriptome and protein level is revealed when relating CD2, CD3ε, CCL19, and CCL5 to TGFβ1 (Figs. 6C and 6D). Up-regulation of these T cell regulated key factors is shown to inhibit TGFβ1 indirectly through IFNγ (which is shown to be up-regulated), which was independently analyzed to occur in tumors of old hosts. One of the multiple roles of TGFβ in late stage tumors is the promotion of loss of growth inhibitory controls that will facilitate evasion of immunosurveillance and immunological escape [48, 49]. Additionally, TGFβ has inhibitory effects on the immune system by directly inhibiting both T cell proliferation and activation, suppressing the cytotoxic T lymphocyte program, suppressing production of IFN, and inhibiting APCs [11, 15, 44, 48, 49]. The inhibition of TGFβ has been shown to directly enhance NK cells, macrophage functions, and T cell activity with the assistance of up-regulation of IFNγ and directly contribute to a T cell mediated inhibition of tumor growth [49]. For old tumor bearing hosts, this interplay between the increase of the key T cell related factors from the spleen with the down-regulation of TGFβ in the tumor, counter the expected age associated immune suppression.

**Figure 7:**
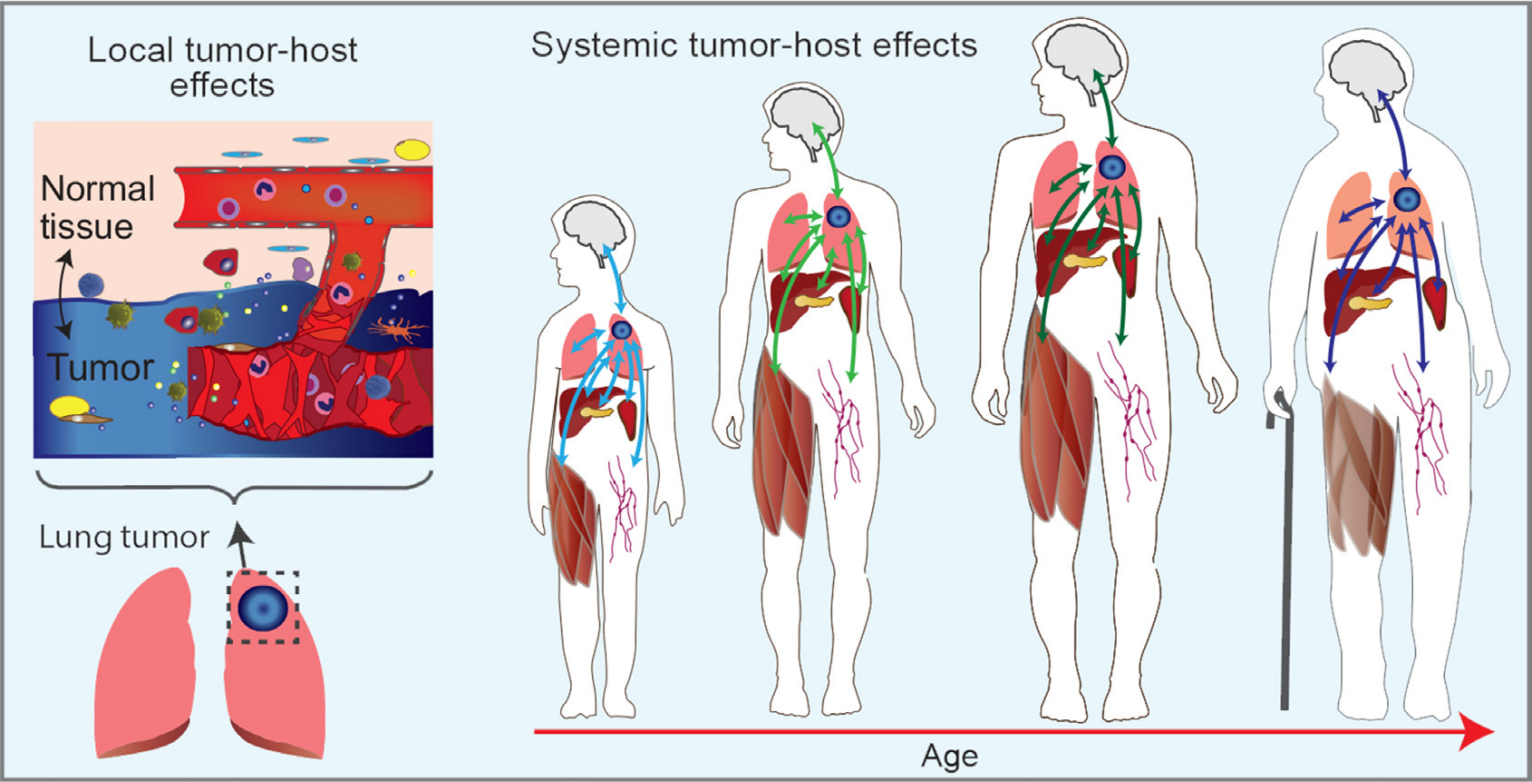
Schematic of systemic tumor-host effects as a function of age

Current models to study aging effects and cancer risk either are observational results from clinical data or limited to only immediate specific effects (i.e. tumor related angiogenesis) of the tumor on the host. The data from these types of studies only reveal a limited view of the cancer risk associated with the host by only observing the system surrounding the immediate tumor at a given time. Tumor burden tends to not only affect the immediate tumor microenvironment (TME), but also impact the host as a whole (Fig. 7). It also orchestrates a dynamic tumor-host evolution that, given the connection between risk and aging, stands to be vastly informed by tracking these events with age. Information is lacking on a systems level both with the entire pathways and networks involved in the TME and also long distance effects to other areas of the host, and is even more deficient where age dependence is concerned. Rather than focusing on certain factors, we have begun to study the whole system (i.e. the host) starting with the effects of the spleen as a function of age with and without tumor burden. By looking as a function of age at global transcription changes that occur in the spleen with and without tumor burden, creates a connected view and map of systemic changes over time. Additionally, introducing more organs will provide a more complete view of how the interconnectivity of all the organs will change as a function age and will in return impact tumor progression (Fig. 7). This data may determine key operators originating from systemic tissues or directly from the presence of a tumor that may be used to improve assessment of cancer risk or treatment as a function of age.

## MATERIALS AND METHODS

### Cell culture

Murine Lewis lung carcinoma (LLC) cells, originally derived from a spontaneous tumor in a C57BL/6 mouse [50], were obtained from American Type Culture Collection (Manassas, VA). The LLC cells were cultured under standard conditions [50] in high glucose DMEM with 10% FBS (Gibco Invitrogen Cell Culture, Carlsbad, CA) and 5% CO_2_.

### Tumor injections

Tumor injections for this study were previously described by Beheshti et al., for the same mice used in a previous study [9]. Scaling of mouse age to human age was accomplished using published criteria [51]. Age comparisons are as follows: mice at 68 (adolescent) and 736 (old) days old are considered equivalent to 17 and 75 year old humans.

### Tissue processing

Tissues to be paraffin-sectioned were placed in 10% formalin, processed by standard protocol [52] placed in cassettes, and paraffin-embedded. Paraffin-embedded tissues were cut into 4 μm slices, placed on positively charged slides (Fisher Scientific, Pittsburg, PA), and stained for hematoxylin and eosin (H&E) stain using standard protocols [52].

### Real-time quantitative PCR

RNA was isolated from tumor tissue in TRIzol (Invitrogen, Carlsbad, CA) using standard methods and homogenized using a Tissue Lyser II (Qiagen, Valencia, CA). Tissue with TRIzol was extracted according to the manufacturer's instruction as was previously reported [8]. Probes for CCL19, CD2, CCL5, and CD3E were commercially available (Applied Biosystems, Carlsbad, CA). Assays were performed with technical duplicates and data was analyzed using the method described by Schmittgen and Livak [53].

### Westerns

Total protein was extracted from snap frozen tissue by standard extraction protocol and homogenized using a Tissue Lyser II (Qiagen) as previously reported [9]. 50μg quantity of total protein from each tissue lysis was separated on a 10% Tricine protein gel (Life Technologies, Carlsbad, CA) at 125V for 1hr and transferred to nitrocellulose membranes (Amersham Biosciences, Pittsburgh, PA) at 22V for 1.5hrs. Membranes were blocked in PBS containing 0.05% Tween 20 and 5% nonfat dry milk for 1hr, and then probed with CD2 and CD3E antibody (Abcam, Cambridge, MA), overnight at 4°C. After washing, membranes were incubated with HRP-conjugated secondary antibody (diluted 1:2000, Santa Cruz Biotechnology, Dallas, TX) for 1hr, room temperature. An ECL Plus detection kit (Amersham Biosciences) was used to visualize bands by chemiluminescence. GAPDH (Santa Cruz Biotechnology) was used as a technical control.

### Gene expression analysis

For genome-wide expression profiling of tumor tissue, Mouse WG-6 bead array chips (Illumina, San Diego, CA) were used. Methods for obtaining gene expression array data were previously reported [8, 54]. For spleen replicates, 10 spleen samples for each condition (adolescent, young adult, middle-aged, and old) from tumor and non-tumor bearing mice for a total of 80 spleen samples were used. The data was corrected through COMBAT batch correction [55] then quantile normalization was applied. Data was imported into MultiExperiment Viewer, MeV [56] for analysis. The statistically significant genes were determined by applying a one-way ANOVA with an adjusted Bonferroni correction for a false discovery rate (FDR) < 0.05 that resulted in a list of 122 significant genes for spleens from tumor bearing mice and 2102 significant genes for spleens from non-tumor bearing mice.

Further pathway analysis of the significant gene lists for spleens from both tumor and non-bearing mice were performed by using a fold change greater than ±1.2 comparing all samples to each other and observing pathway relationships using Ingenuity Pathway Analysis (IPA) software (Ingenuity Systems, Redwood City, CA). Upstream regulator analysis from IPA identified any molecule that affected the expression or function of the measured downstream target genes. This included transcription regulators, growth factors, cytokines, enzymes, transmembrane receptors, and kinases. This analysis uses expected causal effects from the gene expression list compiled in this study and uses a Fisher's test with a comprehensive database of known upstream regulators from the literature to determine significance. The activation state of each upstream regulator from the experimental data set is determined by calculating the z-score (≥ 2, activated or ≤ −2, inhibited). Gene Set Enrichment Analysis (GSEA) [57] was also performed using the entire list of genes and with leading edge analysis. Significant gene sets between age groups were considered with FDR < 0.05. The data discussed in this publication have been deposited in NCBI's Gene Expression Omnibus [58] and are accessible through GEO Series accession number GSE73451 (http://www.ncbi.nlm.nih.gov/geo/query/acc.cgi?acc=GSE73451).

### Statistical analysis

Student's *t*-tests were used for statistical analysis as appropriate. All *p*-values were calculated using two-tailed tests. Differences were considered statistically significant if *p* < 0.05. Error bars in the graphs represent standard error.

## SUPPLEMENTARY MATERIAL TABLES



## References

[1] Baffert F, Thurston G, Rochon-Duck M, Le T, Brekken R, McDonald DM (2004). Age-related changes in vascular endothelial growth factor dependency and angiopoietin-1-induced plasticity of adult blood vessels. Circ Res.

[2] Edelberg JM, Reed MJ (2003). Aging and angiogenesis. Front Biosci.

[3] Campisi J (2005). Senescent cells, tumor suppression, and organismal aging: good citizens, bad neighbors. Cell.

[4] Lu P, Weaver VM, Werb Z (2012). The extracellular matrix: a dynamic niche in cancer progression. J Cell Biol.

[5] Ershler WB (1986). Why tumors grow more slowly in old people. J Natl Cancer Inst.

[6] Enderling H, Hlatky L, Hahnfeldt P (2012). Immunoediting: evidence of the multifaceted role of the immune system in self-metastatic tumor growth. Theor Biol Med Model.

[7] Harding C, Pompei F, Lee EE, Wilson R (2008). Cancer suppression at old age. Cancer Res.

[8] Beheshti A, Sachs RK, Peluso M, Rietman E, Hahnfeldt P, Hlatky L (2013). Age and space irradiation modulate tumor progression: implications for carcinogenesis risk. Radiat Res.

[9] Beheshti A, Benzekry S, McDonald JT, Ma L, Peluso M, Hahnfeldt P, Hlatky L (2015). Host age is a systemic regulator of gene expression impacting cancer progression. Cancer Res.

[10] Lopez-Otin C, Blasco MA, Partridge L, Serrano M, Kroemer G (2013). The hallmarks of aging. Cell.

[11] Bierie B, Moses HL (2006). Tumour microenvironment: TGFbeta: the molecular Jekyll and Hyde of cancer. Nat Rev Cancer.

[12] Langley RR, Fidler IJ (2007). Tumor cell-organ microenvironment interactions in the pathogenesis of cancer metastasis. Endocr Rev.

[13] Cesta MF (2006). Normal structure, function, and histology of the spleen. Toxicol Pathol.

[14] Howcroft TK, Campisi J, Louis GB, Smith MT, Wise B, Wyss-Coray T, Augustine AD, McElhaney JE, Kohanski R, Sierra F (2013). The role of inflammation in age-related disease. Aging (Albany NY).

[15] Bronte V, Pittet MJ (2013). The spleen in local and systemic regulation of immunity. Immunity.

[16] Banerjee M, Sanderson JD, Spencer J, Dunn-Walters DK (2000). Immunohistochemical analysis of ageing human B and T cell populations reveals an age-related decline of CD8 T cells in spleen but not gut-associated lymphoid tissue (GALT). Mech Ageing Dev.

[17] Spoor MS, Radi ZA, Dunstan RW (2008). Characterization of age- and gender-related changes in the spleen and thymus from control cynomolgus macaques used in toxicity studies. Toxicol Pathol.

[18] Mebius RE, Kraal G (2005). Structure and function of the spleen. Nat Rev Immunol.

[19] Meier JM, Alavi A, Iruvuri S, Alzeair S, Parker R, Houseni M, Hernandez-Pampaloni M, Mong A, Torigian DA (2007). Assessment of age-related changes in abdominal organ structure and function with computed tomography and positron emission tomography. Semin Nucl Med.

[20] Cortez-Retamozo V, Etzrodt M, Newton A, Rauch PJ, Chudnovskiy A, Berger C, Ryan RJ, Iwamoto Y, Marinelli B, Gorbatov R, Forghani R, Novobrantseva TI, Koteliansky V, Figueiredo JL, Chen JW, Anderson DG (2012). Origins of tumor-associated macrophages and neutrophils. Proc Natl Acad Sci U S A.

[21] Ugel S, Peranzoni E, Desantis G, Chioda M, Walter S, Weinschenk T, Ochando JC, Cabrelle A, Mandruzzato S, Bronte V (2012). Immune tolerance to tumor antigens occurs in a specialized environment of the spleen. Cell Rep.

[22] Fotiadis C, Zografos G, Aronis K, Troupis TG, Gorgoulis VG, Sechas MN, Skalkeas G (1999). The effect of various types of splenectomy on the development of B-16 melanoma in mice. Anticancer Res.

[23] Schwarz RE, Hiserodt JC (1990). Effects of splenectomy on the development of tumor-specific immunity. J Surg Res.

[24] Wilkins AL, Yang W, Yang JJ (2003). Structural biology of the cell adhesion protein CD2: from molecular recognition to protein folding and design. Curr Protein Pept Sci.

[25] Kuang DM, Zhao Q, Xu J, Yun JP, Wu C, Zheng L (2008). Tumor-educated tolerogenic dendritic cells induce CD3epsilon down-regulation and apoptosis of T cells through oxygen-dependent pathways. J Immunol.

[26] Marques RE, Guabiraba R, Russo RC, Teixeira MM (2013). Targeting CCL5 in inflammation. Expert Opin Ther Targets.

[27] Campisi J (2013). Aging, cellular senescence, and cancer. Annu Rev Physiol.

[28] Gorbunova V, Seluanov A, Mao Z, Hine C (2007). Changes in DNA repair during aging. Nucleic Acids Res.

[29] Lewis DA, Travers JB, Spandau DF (2009). A new paradigm for the role of aging in the development of skin cancer. J Invest Dermatol.

[30] Nakaji M, Yano Y, Ninomiya T, Seo Y, Hamano K, Yoon S, Kasuga M, Teramoto T, Hayashi Y, Yokozaki H (2004). IFN-alpha prevents the growth of pre-neoplastic lesions and inhibits the development of hepatocellular carcinoma in the rat. Carcinogenesis.

[31] Romieu-Mourez R, Solis M, Nardin A, Goubau D, Baron-Bodo V, Lin R, Massie B, Salcedo M, Hiscott J (2006). Distinct roles for IFN regulatory factor (IRF)-3 and IRF-7 in the activation of antitumor properties of human macrophages. Cancer Res.

[32] Bedel R, Thiery-Vuillemin A, Grandclement C, Balland J, Remy-Martin JP, Kantelip B, Pallandre JR, Pivot X, Ferrand C, Tiberghien P, Borg C (2011). Novel role for STAT3 in transcriptional regulation of NK immune cell targeting receptor MICA on cancer cells. Cancer Res.

[33] Bossi F, Tripodo C, Rizzi L, Bulla R, Agostinis C, Guarnotta C, Munaut C, Baldassarre G, Papa G, Zorzet S, Ghebrehiwet B, Ling GS, Botto M, Tedesco F (2014). C1q as a unique player in angiogenesis with therapeutic implication in wound healing. Proc Natl Acad Sci U S A.

[34] Petry F, Botto M, Holtappels R, Walport MJ, Loos M (2001). Reconstitution of the complement function in C1q-deficient (C1qa−/−) mice with wild-type bone marrow cells. J Immunol.

[35] Fermino ML, Dias FC, Lopes CD, Souza MA, Cruz AK, Liu FT, Chammas R, Roque-Barreira MC, Rabinovich GA, Bernardes ES (2013). Galectin-3 negatively regulates the frequency and function of CD4(+) CD25(+) Foxp3(+) regulatory T cells and influences the course of Leishmania major infection. Eur J Immunol.

[36] Gauger KJ, Schneider SS (2014). Tumour supressor secreted frizzled related protein 1 regulates p53-mediated apoptosis. Cell Biol Int.

[37] Yoshida R, Imai T, Hieshima K, Kusuda J, Baba M, Kitaura M, Nishimura M, Kakizaki M, Nomiyama H, Yoshie O (1997). Molecular cloning of a novel human CC chemokine EBI1-ligand chemokine that is a specific functional ligand for EBI1, CCR7. J Biol Chem.

[38] Jensen LJ, Kuhn M, Stark M, Chaffron S, Creevey C, Muller J, Doerks T, Julien P, Roth A, Simonovic M, Bork P, von Mering C (2009). STRING 8--a global view on proteins and their functional interactions in 630 organisms. Nucleic Acids Res.

[39] Lokate M, Stellato RK, Veldhuis WB, Peeters PH, van Gils CH (2013). Age-related changes in mammographic density and breast cancer risk. Am J Epidemiol.

[40] Mueller SN, Germain RN (2009). Stromal cell contributions to the homeostasis and functionality of the immune system. Nat Rev Immunol.

[41] Franceschi C, Bonafe M, Valensin S, Olivieri F, De Luca M, Ottaviani E, De Benedictis G (2000). Inflamm-aging. An evolutionary perspective on immunosenescence. Ann N Y Acad Sci.

[42] Baylis D, Bartlett DB, Patel HP, Roberts HC (2013). Understanding how we age: insights into inflammaging. Longev Healthspan.

[43] Pawelec G, Derhovanessian E, Larbi A (2010). Immunosenescence and cancer. Crit Rev Oncol Hematol.

[44] Malaguarnera L, Cristaldi E, Malaguarnera M (2010). The role of immunity in elderly cancer. Crit Rev Oncol Hematol.

[45] Bouker KB, Skaar TC, Riggins RB, Harburger DS, Fernandez DR, Zwart A, Wang A, Clarke R (2005). Interferon regulatory factor-1 (IRF-1) exhibits tumor suppressor activities in breast cancer associated with caspase activation and induction of apoptosis. Carcinogenesis.

[46] Wall L, Burke F, Barton C, Smyth J, Balkwill F (2003). IFN-gamma induces apoptosis in ovarian cancer cells in vivo and in vitro. Clin Cancer Res.

[47] Crawford K, Stark A, Kitchens B, Sternheim K, Pantazopoulos V, Triantafellow E, Wang Z, Vasir B, Larsen CE, Gabuzda D, Reinherz E, Alper CA (2003). CD2 engagement induces dendritic cell activation: implications for immune surveillance and T-cell activation. Blood.

[48] Thomas DA, Massague J (2005). TGF-beta directly targets cytotoxic T cell functions during tumor evasion of immune surveillance. Cancer Cell.

[49] McEarchern JA, Besselsen DG, Akporiaye ET (1999). Interferon gamma and antisense transforming growth factor beta transgenes synergize to enhance the immunogenicity of a murine mammary carcinoma. Cancer Immunol Immunother.

[50] Bertram JS, Janik P (1980). Establishment of a cloned line of Lewis Lung Carcinoma cells adapted to cell culture. Cancer Lett.

[51] Carnes BA, Grahn D, Hoel D (2003). Mortality of atomic bomb survivors predicted from laboratory animals. Radiat Res.

[52] Lillie RD (1965). Histopathologic technic and practical histochemistry.

[53] Schmittgen TD, Livak KJ (2008). Analyzing real-time PCR data by the comparative C(T) method. Nat Protoc.

[54] Beheshti A, Peluso M, Lamont C, Hahnfeldt P, Hlatky L (2014). Proton irradiation augments the suppression of tumor progression observed with advanced age. Radiat Res.

[55] Johnson WE, Li C, Rabinovic A (2007). Adjusting batch effects in microarray expression data using empirical Bayes methods. Biostatistics.

[56] Saeed AI, Bhagabati NK, Braisted JC, Liang W, Sharov V, Howe EA, Li J, Thiagarajan M, White JA, Quackenbush J (2006). TM4 microarray software suite. Methods Enzymol.

[57] Subramanian A, Tamayo P, Mootha VK, Mukherjee S, Ebert BL, Gillette MA, Paulovich A, Pomeroy SL, Golub TR, Lander ES, Mesirov JP (2005). Gene set enrichment analysis: a knowledge-based approach for interpreting genome-wide expression profiles. Proc Natl Acad Sci U S A.

[58] Edgar R, Domrachev M, Lash AE (2002). Gene Expression Omnibus: NCBI gene expression and hybridization array data repository. Nucleic Acids Res.

