# Supplementary material for: Tumor-host signaling interaction reveals a systemic, age-dependent splenic immune influence on tumor development
Source: Oncotarget. 2015 Oct 21;6(34):35419–32. doi: 10.18632/oncotarget.6214 (PMC4742115; doi:10.18632/oncotarget.6214)
Supplement: Supplementary file 1 [file oncotarget-06-35419-s001.pdf]

Tumor-host signaling interaction reveals a systemic, age-dependent splenic immune influence on tumor development

Supplementary Material

Supplemental Table 1. Complete list of *Gene Set Enrichment Analysis (GSEA) for GO gene sets* in the spleen of LLC tumor bearing C57BL/6 male mice with different age comparisons (Adolescent (A), Young Adult (Y), Middle-Aged (M), and Old (O)). *Leading edge analysis with a FWER < 0.05 determined significant gene sets enriched for each group. The normalized enrichment score (NES) indicates whether the gene set is up- (>0) or down-regulated (<0) for each group determined with the # of leading edge genes (appears in parenthesis).*

Gene Set Enrichment	NES (# of Leading Edge Genes)					
	O vs M	O vs Y	O vs A	M vs Y	M vs A	Y vs A
Cell Activation	1.97 (21)	2.06 (29)	2.11 (20)	2.00 (21)	---	---
Cell Cycle GO 0007049	-2.34 (93)	-2.32 (101)	-2.29 (94)	-1.99 (87)	---	---
Cell Cycle Process	-2.48 (65)	-2.39 (64)	-2.47 (65)	-2.04 (60)	---	---
Chromosome	-2.16 (35)	-2.24 (42)	-2.34 (42)	-2.01 (48)	---	---
Immune Response	2.27 (78)	2.37 (84)	2.45 (61)	2.26 (64)	---	---
Immune System Process	2.26 (74)	2.3 (107)	2.41 (86)	2.18 (92)	---	---
Spindle	-2.05 (10)	-2.01 (10)	-2.09 (10)	-2.18 (7)	---	---
Spindle Pole	-2.03 (18)	-2.00 (17)	-2.11 (17)	-2.03 (13)	---	---
Chemokine Activity	2.03 (10)	2.02 (11)	2.13 (10)	---	---	---
Chemokine Receptor Binding	2.04 (10)	2.02 (11)	2.11 (10)	---	---	---
Cell Cycle Phase	-2.52 (58)	-2.33 (54)	-2.38 (56)	---	---	---
Chromosomal Part	-2.17 (31)	-2.14 (35)	-2.22 (34)	---	---	---
Chromosomepericentric Region	-2.17 (14)	-2.09 (17)	-2.11 (15)	---	---	---
DNA Replication	-2.00 (28)	-1.96 (30)	-2.06 (29)	---	---	---
G1 S Transition Of Mitotic Cell Cycle	-2.13 (12)	-1.97 (11)	-2.01 (11)	---	---	---
Interphase	-2.34 (26)	-2.17 (22)	-2.08 (24)	---	---	---
Interphase Of Mitotic Cell Cycle	-2.29 (27)	-2.25 (23)	-2.18 (25)	---	---	---
Kinetochore	-1.97 (10)	-1.96 (13)	-1.94 (11)	---	---	---
Leukocyte Activation	2.10 (21)	2.08 (28)	2.15 (20)	---	---	---
Lymphocyte Activation	2.03 (19)	2.08 (26)	2.10 (18)	---	---	---
M Phase	-2.32 (34)	-2.18 (36)	-2.29 (34)	---	---	---
M Phase Of Mitotic Cell Cycle	-2.28 (39)	-2.18 (42)	-2.26 (42)	---	---	---
Mitosis	-2.24 (32)	-2.15 (35)	-2.26 (33)	---	---	---
Mitotic Cell Cycle	-2.52 (58)	-2.37 (52)	-2.41 (54)	---	---	---
Regulation Of Immune System Process	2.12 (21)	1.92 (23)	1.96 (15)	---	---	---
T Cell Activation	2.16 (14)	1.98 (20)	2.01 (12)	---	---	---
Humoral Immune Response	---	2.10 (11)	2.13 (12)	2.29 (17)	2.22 (16)	---
MRNA Metabolic Process	---	-1.94 (27)	-2.05 (27)	-2.02 (25)	-1.98 (20)	---
MRNA Processing GO 0006397	---	-2.00 (25)	-2.09 (25)	-2.10 (22)	-2.02 (18)	---
Condensed Chromosome	---	-1.95 (14)	-2.06 (13)	-1.98 (16)	---	---
Microtubule Cytoskeleton	---	-2.01 (41)	-2.08 (35)	-2.05 (30)	---	---
Nuclear Part	---	-2.07 (162)	-2.20 (165)	-1.94 (167)	---	---
Nuclear Pore	---	-2.05 (15)	-1.91 (17)	-2.03 (21)	---	---
RNA Processing	---	-1.97 (54)	-2.00 (52)	-1.95 (48)	---	---
Cellular Defense Response	---	2.12 (10)	1.99 (9)	---	---	---

Chromosome Organization And Biogenesis	---	-1.88 (41)	-2.08 (44)	---	---	---
Envelope	---	-1.96 (54)	-1.95 (62)	---	---	---
Microtubule	---	-1.90 (16)	-1.94 (14)	---	---	---
Microtubule Cytoskeleton Organization And Biogenesis	---	-2.03 (11)	-2.02 (12)	---	---	---
Mitochondrial Matrix	---	-1.89 (24)	-1.99 (18)	---	---	---
Mitochondrion	---	-1.88 (105)	-2.04 (115)	---	---	---
Nuclear Membrane Part	---	-2.12 (21)	-2.03 (20)	---	---	---
Organelle Envelope	---	-1.98 (54)	-1.95 (62)	---	---	---
RNA Splicing	---	-1.98 (26)	-1.99 (27)	---	---	---
Blood Coagulation	-2.05 (12)	---	---	2.08 (10)	---	---
Coagulation	-1.98 (12)	---	---	2.06 (10)	---	---
Hemostasis	-2.01 (13)	---	---	1.94 (10)	---	---
Intracellular Receptor Mediated Signaling Pathway	2.00 (12)	---	---	---	-1.98 (11)	---
Cell Cycle Checkpoint GO 0000075	-1.96 (22)	---	---	---	---	---
Positive Regulation Of Immune System Process	2.18 (19)	---	---	---	---	---
Positive Regulation Of Lymphocyte Activation	2.01 (9)	---	---	---	---	---
Positive Regulation Of Multicellular Organismal Process	1.95 (23)	---	---	---	---	---
Positive Regulation Of T Cell Activation	2.00 (8)	---	---	---	---	---
Regulation Of Body Fluid Levels	-2.08 (16)	---	---	---	---	---
Regulation Of Cell Cycle	-1.97 (39)	---	---	---	---	---
Regulation Of Lymphocyte Activation	1.96 (10)	---	---	---	---	---
Regulation Of T Cell Activation	2.15 (9)	---	---	---	---	---
Pore Complex	---	-1.99 (18)	---	-2.00 (23)	---	---
Adaptive Immune Response	---	1.92 (18)	---	---	---	---
Behavior	---	1.94 (29)	---	---	---	---
Chromatin Binding	---	-1.99 (16)	---	---	---	---
G Protein Coupled Receptor Binding	---	1.91 (12)	---	---	---	---
Locomotory Behavior	---	1.98 (26)	---	---	---	---
Mitochondrial Membrane Part	---	-1.98 (20)	---	---	---	---
Nuclear Envelope	---	-1.91 (24)	---	---	---	---
Nuclear Membrane	---	-1.94 (19)	---	---	---	---
Organelle Inner Membrane	---	-1.90 (22)	---	---	---	---
Protein Folding	---	-1.93 (20)	---	---	---	---
Receptor Binding	---	1.93 (76)	---	---	---	---
Nucleolus	---	---	-1.93 (39)	---	-1.98 (32)	---
Axonogenesis	---	---	1.97 (7)	---	---	---
DNA Dependent Atpase Activity	---	---	-1.94 (10)	---	---	---
DNA Dependent DNA Replication	---	---	-1.99 (18)	---	---	---
DNA Metabolic Process	---	---	-2.10 (72)	---	---	---
DNA Repair	---	---	-2.10 (39)	---	---	---
Membrane Enclosed Lumen	---	---	-2.01 (125)	---	---	---
Mitochondrial Lumen	---	---	-1.96 (18)	---	---	---
Mitochondrial Part	---	---	-1.96 (59)	---	---	---

Neurite Development	---	---	1.95 (9)	---	---	---
Neuron Development	---	---	1.97 (11)	---	---	---
Nuclear Chromosome	---	---	-1.99 (15)	---	---	---
Nuclear Lumen	---	---	-2.05 (108)	---	---	---
Nucleoplasm	---	---	-1.93 (71)	---	---	---
Organellar Ribosome	---	---	-1.96 (11)	---	---	---
Organelle Lumen	---	---	-1.99 (125)	---	---	---
Positive Regulation Of I Kappab Kinase NF Kappab Cascade	---	---	1.94 (33)	---	---	---
Response To DNA Damage Stimulus	---	---	-1.99 (48)	---	---	---
Response To Endogenous Stimulus	---	---	-1.94 (55)	---	---	---
Ribonucleoprotein Complex Biogenesis And Assembly	---	---	-1.95 (26)	---	---	---
Centrosome	---	---	---	-2.02 (19)	---	---
Defense Response	---	---	---	2.09 (54)	---	---
Inflammatory Response	---	---	---	2.09 (31)	---	---
Microtubule Organizing Center	---	---	---	-2.01 (20)	---	---
Response To External Stimulus	---	---	---	1.95 (62)	---	---
Response To Wounding	---	---	---	2.11 (42)	---	---
Ribonucleoprotein Complex	---	---	---	-1.98 (48)	---	---
Wound Healing	---	---	---	1.98 (10)	---	---
Membrane Fusion	---	---	---	---	2.02 (10)	---
Steroid Hormone Receptor Signaling Pathway	---	---	---	---	-1.98 (11)	---

Supplemental Table 2. Complete list of Gene Set Enrichment Analysis (GSEA) for GO gene sets in the spleen of non-tumor bearing C57BL/6 male mice with different age comparisons (Adolescent (A), Young Adult (Y), Middle-Aged (M), and Old (O)). Leading edge analysis with a FDR < 0.05 determined significant gene sets enriched for each group. The normalized enrichment score (NES) indicates whether the gene set is up- (>0) or down-regulated (<0) for each group determined with the # of leading edge genes (appears in parenthesis).

Gene Set Enrichment	NES (# of Leading Edge Genes)					
	O vs M	O vs Y	O vs A	M vs Y	M vs A	Y vs A
Carbohydrate Binding	1.74 (19)	-2.02 (18)	1.96 (15)	---	---	---
Extracellular Matrix	1.77 (23)	-2.20 (16)	1.97 (24)	---	---	---
Extracellular Region	1.67 (116)	-2.24 (74)	2.28 (53)	---	---	---
Glycosaminoglycan Binding	1.83 (12)	-1.79 (11)	1.86 (10)	---	---	---
Heparin Binding	1.77 (11)	-1.60 (9)	1.69 (7)	---	---	---
Pattern Binding	1.82 (14)	-1.85 (16)	2.03 (13)	---	---	---
Polysaccharide Binding	1.89 (12)	-1.85 (11)	1.77 (10)	---	---	---
Proteinaceous Extracellular Matrix	1.72 (25)	-2.17 (28)	2.01 (27)	---	---	---
Response To Oxidative Stress	1.80 (14)	-1.65 (13)	2.12 (20)	---	---	1.71 (18)
Structural Constituent Of Ribosome	2.21 (38)	1.83 (34)	-2.00 (41)	2.31 (47)	-2.29 (45)	---
Mitochondrial Inner Membrane	1.85 (25)	1.60 (21)	---	1.96 (28)	-1.78 (27)	1.81 (25)
Mitochondrial Lumen	1.85 (17)	1.87 (25)	---	1.92 (23)	-1.84 (18)	1.69 (21)
Mitochondrial Matrix	1.82 (17)	1.86 (25)	---	1.94 (23)	-1.86 (18)	1.66 (21)
Mitochondrial Membrane Part	1.96 (29)	1.55 (17)	---	2.00 (33)	-1.81 (31)	1.82 (31)
Mitochondrial Part	1.89 (49)	1.82 (56)	---	2.02 (59)	-1.94 (51)	1.78 (51)
Mitochondrial Ribosome	1.87 (13)	1.87 (14)	---	1.89 (13)	-1.83 (13)	1.98 (13)
Mitochondrion	1.76 (82)	1.64 (104)	---	1.91 (102)	-1.70 (91)	1.86 (115)
Organellar Ribosome	1.88 (13)	1.88 (14)	---	1.88 (13)	-1.88 (13)	1.96 (13)
Organelle Inner Membrane	1.70 (26)	1.87 (25)	---	1.91 (34)	-1.70 (29)	1.85 (27)
Ribosomal Subunit	1.89 (12)	1.87 (13)	---	1.89 (13)	-1.85 (12)	1.90 (11)
Ribosome	1.97 (19)	1.59 (20)	---	2.07 (22)	-2.06 (19)	---
Translation Regulator Activity	-1.79 (23)	1.68 (19)	---	---	---	---
Oxygen And Reactive Oxygen Species Metabolic Process	1.90 (8)	---	1.63 (7)	1.82 (8)	-1.69 (7)	1.80 (6)
Glutathione Transferase Activity	1.69 (13)	---	1.64 (8)	---	---	---
Actin Filament Binding	---	-1.69 (7)	1.71 (9)	---	---	---
Amine Metabolic Process	---	-1.71 (27)	1.80 (37)	---	---	---
Anatomical Structure Formation	---	-1.79 (13)	1.84 (13)	---	---	---
Anatomical Structure Morphogenesis	---	-1.90 (74)	1.75 (67)	---	---	---
Angiogenesis	---	-1.67 (10)	1.81 (12)	---	---	---
Basal Lamina	---	-1.76 (9)	1.78 (9)	---	---	---
Basement Membrane	---	-1.92 (14)	1.83 (17)	---	---	---
Calcium Ion Binding	---	-1.62 (17)	1.63 (23)	---	---	---
Calmodulin Binding	---	-1.85 (10)	1.69 (12)	---	---	---
Cation Binding	---	-1.61 (36)	1.70 (41)	---	---	---
Cell Migration	---	-2.14 (26)	1.76 (21)	---	---	---
Cell Surface	---	-1.75 (23)	1.66 (21)	---	---	---
Chemokine Receptor Binding	---	-1.97 (11)	1.64 (11)	---	---	---
Cytokine Activity	---	-1.87 (18)	1.64 (20)	---	---	---
Endoplasmic Reticulum	---	-1.81 (67)	1.68 (70)	---	---	---
Extracellular Matrix Part	---	-2.05 (29)	1.90 (28)	---	---	---
Extracellular Matrix Structural	---	-1.67 (9)	1.76 (7)	---	---	---

Constituent						
Extracellular Region Part	---	-2.24 (98)	2.16 (76)	---	---	---
Extracellular Space	---	-2.03 (48)	2.09 (36)	---	---	---
Growth Factor Activity	---	-1.82 (14)	1.70 (12)	---	---	---
Inflammatory Response	---	-1.90 (42)	1.65 (29)	---	---	---
Intercellular Junction	---	-1.70 (8)	1.69 (9)	---	---	---
Locomotory Behavior	---	-1.94 (19)	1.67 (21)	---	---	---
Lysosome	---	-1.66 (23)	1.70 (14)	---	---	---
Lytic Vacuole	---	-1.65 (23)	1.68 (14)	---	---	---
Membrane Organization And Biogenesis	---	-1.88 (41)	1.69 (34)	---	---	---
Multi Organism Process	---	-1.85 (25)	1.74 (23)	---	---	---
Neurite Development	---	-1.82 (13)	1.63 (11)	---	---	---
Neuron Development	---	-1.92 (13)	1.75 (13)	---	---	---
Nitrogen Compound Metabolic Process	---	-1.62 (28)	1.78 (44)	---	---	---
Oxidoreductase Activity GO 0016705	---	-1.71 (8)	1.63 (71)	---	---	---
Protease Inhibitor Activity	---	-2.18 (11)	1.92 (11)	---	---	---
Response To ExteRNAl Stimulus	---	-1.75 (73)	1.76 (74)	---	---	---
Response To Other Organism	---	-1.64 (19)	1.73 (16)	---	---	---
Response To Wounding	---	-1.79 (55)	1.76 (44)	---	---	---
Serine Type Endopeptidase Inhibitor Activity	---	-1.97 (5)	1.72 (5)	---	---	---
Skeletal Development	---	-2.07 (27)	1.74 (21)	---	---	---
Synapse	---	-1.64 (7)	1.84 (9)	---	---	---
Transmembrane Receptor Protein Kinase Activity	---	-1.97 (12)	1.81 (13)	---	---	---
Transmembrane Receptor Protein Tyrosine Kinase Activity	---	-1.94 (11)	1.78 (12)	---	---	---
Transport Vesicle	---	-1.63 (9)	1.63 (10)	---	---	---
Vacuole	---	-1.69 (25)	1.68 (15)	---	---	---
Vesicle Mediated Transport	---	-1.95 (53)	1.73 (33)	---	---	---
Mitochondrial Membrane	1.73 (26)	---	---	1.83 (25)	-1.70 (26)	1.76 (24)
Mitochondrial Respiratory Chain	1.77 (13)	---	---	1.75 (11)	-1.66 (13)	1.83 (12)
Mitochondrial Envelope	1.68 (30)	---	---	1.80 (32)	-1.71 (33)	---
Structural Molecule Activity	1.83 (64)	---	---	1.91 (68)	-2.10 (60)	---
Ubiquitin Cycle	-1.92 (23)	---	---	---	1.85 (21)	---
Protein Modification By Small Protein Conjugation	-1.87 (20)	---	---	---	---	---
Protein Ubiquitination	-1.87 (19)	---	---	---	---	---
Ribonucleoprotein Complex	---	2.11 (31)	---	1.83 (48)	-1.81 (40)	---
Small Nuclear Ribonucleoprotein Complex	---	1.82 (7)	---	1.67 (9)	-1.67 (9)	---
Cell Cycle GO 0007049	---	2.16 (110)	---	---	---	2.07 (93)
Cell Cycle Phase	---	2.22 (67)	---	---	---	2.11 (56)
Cell Cycle Process	---	2.31 (78)	---	---	---	2.19 (66)
Centrosome	---	1.89 (22)	---	---	---	1.84 (18)
Chromatin Binding	---	2.25 (17)	---	---	---	1.90 (14)
Chromosomal Part	---	2.28 (34)	---	---	---	1.86 (28)
Chromosome	---	2.32 (44)	---	---	---	1.98 (18)
Chromosome Segregation	---	1.91 (43)	---	---	---	1.94 (42)
Chromosomepericentric Region	---	2.08 (17)	---	---	---	1.71 (13)
Condensed Chromosome	---	1.93 (15)	---	---	---	1.89 (14)

DNA Dependent DNA Replication	---	2.10 (15)	---	---	---	1.81 (13)
DNA Metabolic Process	---	1.97 (98)	---	---	---	1.86 (74)
DNA Packaging	---	1.91 (8)	---	---	---	1.70 (6)
DNA Recombination	---	1.58 (18)	---	---	---	1.65 (13)
DNA Repair	---	1.95 (55)	---	---	---	1.70 (40)
DNA Replication	---	2.21 (29)	---	---	---	1.95 (27)
Envelope	---	1.79 (64)	---	---	---	1.82 (63)
G1 S Transition Of Mitotic Cell Cycle	---	1.98 (16)	---	---	---	1.97 (11)
Interphase	---	2.02 (24)	---	---	---	1.90 (25)
Interphase Of Mitotic Cell Cycle	---	2.05 (25)	---	---	---	1.84 (27)
M Phase	---	2.08 (39)	---	---	---	2.02 (27)
M Phase Of Mitotic Cell Cycle	---	2.04 (47)	---	---	---	1.98 (32)
Microtubule Cytoskeleton	---	1.85 (32)	---	---	---	1.92 (14)
Microtubule Cytoskeleton Organization And Biogenesis	---	1.84 (8)	---	---	---	2.03 (32)
Microtubule Organizing Center	---	1.83 (23)	---	---	---	1.86 (7)
Microtubule Organizing Center Part	---	1.60 (11)	---	---	---	1.76 (18)
Mitosis	---	2.08 (38)	---	---	---	1.94 (23)
Mitotic Cell Cycle	---	2.19 (63)	---	---	---	2.06 (52)
Nuclear Envelope	---	1.67 (27)	---	---	---	1.67 (19)
Nuclear Membrane	---	1.93 (26)	---	---	---	1.80 (19)
Nuclear Membrane Part	---	2.07 (24)	---	---	---	1.94 (20)
Nuclear Pore	---	2.00 (22)	---	---	---	1.92 (15)
Nucleobasenucleoside And Nucleotide Metabolic Process	---	1.57 (17)	---	---	---	1.69 (17)
Organelle Envelope	---	1.79 (64)	---	---	---	1.82 (63)
Spindle	---	2.04 (8)	---	---	---	2.01 (13)
Unfolded Protein Binding	---	1.83 (15)	---	---	---	1.74 (17)
Actin Cytoskeleton Organization And Biogenesis	---	-1.68 (33)	---	---	---	---
Actin Filament Based Process	---	-1.73 (37)	---	---	---	---
Adaptive Immune Response	---	-1.66 (10)	---	---	---	---
Adaptive Immune Response GO 0002460	---	-1.61 (11)	---	---	---	---
Aerobic Respiration	---	1.56 (5)	---	---	---	---
Amine Catabolic Process	---	-1.66 (9)	---	---	---	---
Amino Acid Catabolic Process	---	-1.64 (8)	---	---	---	---
ATP Dependent Helicase Activity	---	1.89 (12)	---	---	---	---
ATPase Activity	---	1.84 (31)	---	---	---	---
ATPase Activity Coupled	---	1.8 (31)	---	---	---	---
Axon Guidance	---	-1.86 (5)	---	---	---	---
Axonogenesis	---	-1.90 (8)	---	---	---	---
Base Excision Repair	---	1.60 (8)	---	---	---	---
Behavior	---	-1.85 (21)	---	---	---	---
Caspase Activation	---	-1.66 (6)	---	---	---	---
Cation Homeostasis	---	-1.73 (17)	---	---	---	---
Cell Activation	---	-1.62 (22)	---	---	---	---
Cell Cell Adhesion	---	-1.70 (15)	---	---	---	---
Cell Cell Signaling	---	-1.67 (51)	---	---	---	---
Cell Cycle Checkpoint GO 0000075	---	1.61 (23)	---	---	---	---
Cell Division	---	1.71 (7)	---	---	---	---

Cell Projection	---	-1.83 (27)	---	---	---	---
Cellular Cation Homeostasis	---	-1.69 (16)	---	---	---	---
Cellular Defense Response	---	-1.66 (10)	---	---	---	---
Cellular Morphogenesis During Differentiation	---	-1.79 (9)	---	---	---	---
Cellular Respiration	---	1.58 (9)	---	---	---	---
Chemical Homeostasis	---	-1.62 (28)	---	---	---	---
Chemokine Activity	---	-1.97 (11)	---	---	---	---
Chromatin	---	1.82 (15)	---	---	---	---
Chromatin Assembly Or Disassembly	---	1.76 (16)	---	---	---	---
Chromatin Modification	---	1.56 (8)	---	---	---	---
Chromatin Remodeling	---	1.62 (9)	---	---	---	---
Chromosome Organization And Biogenesis	---	2.09 (15)	---	---	---	---
Collagen	---	-1.82 (10)	---	---	---	---
Condensed Nuclear Chromosome	---	1.72 (8)	---	---	---	---
Cytokine Biosynthetic Process	---	-1.76 (13)	---	---	---	---
Cytokine Metabolic Process	---	-1.76 (13)	---	---	---	---
Cytokine Production	---	-1.81 (20)	---	---	---	---
Cytoskeletal Part	---	1.71 (53)	---	---	---	---
Cytoskeleton Dependent Intracellular Transport	---	-1.62 (7)	---	---	---	---
Defense Response	---	-1.73 (61)	---	---	---	---
DNA Dependent Atpase Activity	---	2.01 (9)	---	---	---	---
DNA Helicase Activity	---	1.74 (13)	---	---	---	---
DNA Polymerase Activity	---	1.59 (8)	---	---	---	---
Double Strand Break Repair	---	1.55 (5)	---	---	---	---
Double Stranded DNA Binding	---	2.09 (13)	---	---	---	---
Drug Binding	---	1.65 (5)	---	---	---	---
Early Endosome	---	-1.61 (5)	---	---	---	---
Enzyme Inhibitor Activity	---	-1.63 (28)	---	---	---	---
Enzyme Linked Receptor Protein Signaling Pathway	---	-1.87 (38)	---	---	---	---
Enzyme Regulator Activity	---	-1.65 (70)	---	---	---	---
ER Golgi Intermediate Compartment	---	-1.70 (8)	---	---	---	---
Establishment And Or Maintenance Of Chromatin Architecture	---	1.94 (29)	---	---	---	---
Extrinsic To Membrane	---	-1.62 (6)	---	---	---	---
Female Pregnancy	---	-1.71 (7)	---	---	---	---
G Protein Coupled Receptor Binding	---	-1.84 (10)	---	---	---	---
Golgi Apparatus	---	-1.62 (49)	---	---	---	---
Golgi Vesicle Transport	---	-1.63 (14)	---	---	---	---
Gtpase Activator Activity	---	-1.69 (17)	---	---	---	---
Helicase Activity	---	1.84 (21)	---	---	---	---
Hormone Receptor Binding	---	1.60 (8)	---	---	---	---
Hydrolase Activity Hydrolyzing O Glycosyl Compounds	---	-1.71 (11)	---	---	---	---
I Kappab Kinase Nf Kappab Cascade	---	-1.84 (30)	---	---	---	---
Immune Response	---	-1.85 (44)	---	---	---	---
Immune System Process	---	-1.83 (64)	---	---	---	---
Kinetochore	---	1.91 (13)	---	---	---	---
L Amino Acid Transmembrane	---	-1.63 (6)	---	---	---	---

Transporter Activity						
Leading Edge	---	-1.78 (16)	---	---	---	---
Ligase Activity	---	1.64 (18)	---	---	---	---
Ligase Activity Forming Carbon Nitrogen Bonds	---	1.56 (30)	---	---	---	---
Lipase Activity	---	-1.71 (11)	---	---	---	---
Lipid Transport	---	-1.62 (9)	---	---	---	---
Meiotic Cell Cycle	---	1.79 (12)	---	---	---	---
Membrane Enclosed Lumen	---	2.10 (160)	---	---	---	---
Microtubule	---	1.68 (15)	---	---	---	---
mRNA Metabolic Process	---	2.12 (28)	---	---	---	---
mRNA Processing GO 0006397	---	2.19 (25)	---	---	---	---
Muscle Development	---	-1.76 (36)	---	---	---	---
Nuclear Body	---	1.71 (11)	---	---	---	---
Nuclear Chromosome	---	1.92 (10)	---	---	---	---
Nuclear Chromosome Part	---	1.92 (16)	---	---	---	---
Nuclear Export	---	1.85 (18)	---	---	---	---
Nuclear Lumen	---	2.08 (104)	---	---	---	---
Nuclear Organization And Biogenesis	---	1.64 (6)	---	---	---	---
Nuclear Part	---	2.25 (171)	---	---	---	---
Nucleobasenucleosidenucleotide And Nucleic Acid Transport	---	1.85 (17)	---	---	---	---
Nucleolar Part	---	1.57 (5)	---	---	---	---
Nucleolus	---	2.03 (36)	---	---	---	---
Nucleoplasm	---	1.94 (60)	---	---	---	---
Nucleoplasm Part	---	1.85 (75)	---	---	---	---
Nucleotidyltransferase Activity	---	1.71 (19)	---	---	---	---
Organ Morphogenesis	---	-1.75 (29)	---	---	---	---
Organelle Lumen	---	2.08 (160)	---	---	---	---
Peptidyl Tyrosine Modification	---	-1.69 (8)	---	---	---	---
Peptidyl Tyrosine Phosphorylation	---	-1.62 (7)	---	---	---	---
Phospholipase Activity	---	-1.76 (10)	---	---	---	---
Polysaccharide Metabolic Process	---	-1.75 (4)	---	---	---	---
Pore Complex	---	2.12 (25)	---	---	---	---
Positive Regulation Of Caspase Activity	---	-1.62 (9)	---	---	---	---
Positive Regulation Of I Kappab Kinase Nf Kappab Cascade	---	-1.80 (25)	---	---	---	---
Positive Regulation Of Signal Transduction	---	-1.74 (30)	---	---	---	---
Proteasome Complex	---	1.58 (9)	---	---	---	---
Protein Folding	---	1.60 (16)	---	---	---	---
Protein RNA Complex Assembly	---	1.89 (26)	---	---	---	---
Protein Tyrosine Kinase Activity	---	-1.81 (17)	---	---	---	---
Ras Protein Signal Transduction	---	-1.69 (25)	---	---	---	---
Receptor Binding	---	-1.85 (66)	---	---	---	---
Receptor Mediated Endocytosis	---	-1.69 (11)	---	---	---	---
Regulation Of Cell Cycle	---	1.70 (64)	---	---	---	---
Regulation Of I Kappab Kinase Nf Kappab Cascade	---	-1.82 (26)	---	---	---	---
Regulation Of Mitosis	---	1.70 (18)	---	---	---	---
Regulation Of Signal Transduction	---	-1.86 (58)	---	---	---	---

Replication Fork	---	2.06 (13)	---	---	---	---
Response To DNA Damage Stimulus	---	1.89 (68)	---	---	---	---
Response To Endogenous Stimulus	---	1.85 (76)	---	---	---	---
RHO GTPase Activator Activity	---	-1.71 (6)	---	---	---	---
Ribonuclease Activity	---	1.79 (11)	---	---	---	---
Ribonucleoprotein Complex Biogenesis And Assembly	---	1.93 (49)	---	---	---	---
RNA Binding	---	1.82 (69)	---	---	---	---
RNA Export From Nucleus	---	1.86 (13)	---	---	---	---
RNA Helicase Activity	---	1.59 (10)	---	---	---	---
RNA Processing	---	2.25 (59)	---	---	---	---
RNA Splicing	---	2.23 (31)	---	---	---	---
RNA Splicingvia Transesterification Reactions	---	1.73 (10)	---	---	---	---
Ruffle	---	-1.76 (10)	---	---	---	---
Secretion By Cell	---	-1.75 (30)	---	---	---	---
Secretory Pathway	---	-1.63 (20)	---	---	---	---
Single Stranded DNA Binding	---	1.86 (12)	---	---	---	---
Small GTPase Mediated Signal Transduction	---	-1.64 (30)	---	---	---	---
Sodium Channel Activity	---	-1.63 (2)	---	---	---	---
Spindle Pole	---	1.70 (16)	---	---	---	---
Spliceosome	---	2.01 (18)	---	---	---	---
Structure Specific DNA Binding	---	2.20 (21)	---	---	---	---
Sugar Binding	---	-1.62 (8)	---	---	---	---
Thyroid Hormone Receptor Binding	---	1.59 (7)	---	---	---	---
Transforming Growth Factor Beta Receptor Signaling Pathway	---	-2.06 (18)	---	---	---	---
Translation Factor Activity Nucleic Acid Binding	---	1.65 (19)	---	---	---	---
Translation Initiation Factor Activity	---	1.70 (13)	---	---	---	---
Transmembrane Receptor Activity	---	-1.72 (66)	---	---	---	---
Transmembrane Receptor Protein Serine Threonine Kinase Signaling Pathway	---	-2.08 (22)	---	---	---	---
Vasculature Development	---	-1.67 (12)	---	---	---	---
Blood Coagulation	---	---	1.92 (11)	---	---	1.99 (13)
Coagulation	---	---	1.91 (11)	---	---	1.93 (13)
Coenzyme Metabolic Process	---	---	1.62 (12)	---	---	1.80 (15)
Cofactor Biosynthetic Process	---	---	1.75 (10)	---	---	1.77 (11)
Cofactor Metabolic Process	---	---	1.90 (22)	---	---	2.01 (26)
Contractile Fiber	---	---	1.79 (6)	---	---	1.79 (7)
Contractile Fiber Part	---	---	1.76 (7)	---	---	1.71 (8)
Defense Response To Bacterium	---	---	1.82 (7)	---	---	1.64 (5)
Hemostasis	---	---	1.92 (12)	---	---	1.98 (11)
Heterocycle Metabolic Process	---	---	1.91 (11)	---	---	1.92 (10)
Metalloendopeptidase Activity	---	---	1.80 (12)	---	---	1.75 (9)
Oxidoreductase Activity	---	---	1.91 (14)	---	---	1.71 (11)
Oxidoreductase Activity Acting On NADH Or NADPH	---	---	1.74 (9)	---	---	1.86 (12)
Regulation Of Body Fluid Levels	---	---	1.91 (13)	---	---	2.03 (12)
Response To Bacterium	---	---	1.93 (9)	---	---	1.65 (6)

Wound Healing	---	---	1.73 (11)	---	---	1.73 (10)
Amine Transmembrane Transporter Activity	---	---	1.62 (8)	---	---	---
Amino Acid And Derivative Metabolic Process	---	---	1.74 (25)	---	---	---
Amino Acid Metabolic Process	---	---	1.72 (21)	---	---	---
Amino Sugar Metabolic Process	---	---	1.64 (8)	---	---	---
Antioxidant Activity	---	---	1.81 (9)	---	---	---
Carboxylesterase Activity	---	---	1.64 (9)	---	---	---
Carboxylic Acid Metabolic Process	---	---	1.88 (50)	---	---	---
Carboxylic Acid Transmembrane Transporter Activity	---	---	1.65 (9)	---	---	---
Cell Maturation	---	---	1.68 (8)	---	---	---
Clathrin Coated Vesicle	---	---	1.65 (12)	---	---	---
Coated Vesicle	---	---	1.69 (15)	---	---	---
Cytoplasmic Membrane Bound Vesicle	---	---	1.65 (31)	---	---	---
Cytoplasmic Vesicle	---	---	1.73 (9)	---	---	---
Cytoplasmic Vesicle Part	---	---	1.61 (33)	---	---	---
Developmental Maturation	---	---	1.65 (8)	---	---	---
Electron Transport GO 0006118	---	---	1.71 (16)	---	---	---
Fatty Acid Metabolic Process	---	---	1.73 (17)	---	---	---
Ion Binding	---	---	1.61 (58)	---	---	---
Membrane Bound Vesicle	---	---	1.64 (31)	---	---	---
Metallopeptidase Activity	---	---	1.81 (12)	---	---	---
Monocarboxylic Acid Metabolic Process	---	---	1.76 (23)	---	---	---
Myofibril	---	---	1.69 (5)	---	---	---
Organic Acid Metabolic Process	---	---	1.82 (51)	---	---	---
Organic Acid Transmembrane Transporter Activity	---	---	1.64 (9)	---	---	---
Pigment Biosynthetic Process	---	---	1.63 (6)	---	---	---
Pigment Metabolic Process	---	---	1.74 (7)	---	---	---
Regulation Of Angiogenesis	---	---	1.62 (6)	---	---	---
Regulation Of Biological Quality	---	---	1.63 (86)	---	---	---
Regulation Of Neurotransmitter Levels	---	---	1.67 (5)	---	---	---
Response To Chemical Stimulus	---	---	1.64 (61)	---	---	---
Secondary Metabolic Process	---	---	1.86 (10)	---	---	---
Vesicle	---	---	1.69 (42)	---	---	---
DNA Directed RNA Polymeraseii Holoenzyme	---	---	---	---	-1.70 (17)	---
Induction Of Apoptosis By Extracellular Signals	---	---	---	---	-1.67 (9)	---
Mediator Complex	---	---	---	---	-1.68 (7)	---
Exonuclease Activity	---	---	---	---	---	1.76 (7)
Microtubule Organizing Center Organization And Biogenesis	---	---	---	---	---	1.71 (8)
Motor Activity	---	---	---	---	---	1.75 (6)
Nucleotide Metabolic Process	---	---	---	---	---	1.89 (15)
Organelle Membrane	---	---	---	---	---	1.68 (89)
Oxidoreductase Activity Acting On The CH CH Group Of Donors	---	---	---	---	---	1.68 (63)
Secretory Granule	---	---	---	---	---	1.64 (7)
Tubulin Binding	---	---	---	---	---	1.64 (11)

Vesicle Membrane	---	---	---	---	---	1.63 (12)

Supplemental Table 3. Biofunctions predicted to be activated or inhibited in the spleen of LLC tumor bearing C57BL/6 male mice with different age comparisons (Adolescent (A), Young Adult (Y), Middle-Aged (M), and Old (O)) obtained through the use of Ingenuity Pathway Analysis (IPA) software. The predicted activation state of each of these functions is predicted to be increased (z-score ≥ 2) or decrease (z-score ≤ -2) by the use of the regulation z-score which indicates the degree of biofunction activity. The values in parenthesis indicate the number of genes IPA used in the prediction.

IPA Biofunctions Category	Function	Functions Annotation	Activation Z-Score (# of Genes)					
			O vs M	O vs Y	O vs A	M vs Y	M vs A	Y vs A
Cell-To-Cell Signaling and Interaction and Tissue Development	adhesion	adhesion of blood cells	2.073 (8)	2.073 (8)	2.073 (8)	---	---	---
Hematological System Development and Function, Cellular Movement, Immune Cell Trafficking, and Inflammatory Response	cell movement	cell movement of phagocytes	2.029 (7)	2.029 (7)	2.029 (7)	---	---	---
Hematological System Development and Function, Cellular Movement, and Immune Cell Trafficking	migration	migration of antigen presenting cells and phagocytes	2.191 (5)	2.191 (5)	2.191 (5)	---	---	---
Inflammatory Response	migration	migration of phagocytes	2.190 (5)	2.190 (5)	2.190 (5)	---	---	---
Cell Death and Survival	apoptosis	apoptosis of lymphocytes	-2.205 (6)	-2.205 (6)	-2.205 (6)	---	---	---
Cell Death and Survival	apoptosis	apoptosis of leukocytes	-2.612 (7)	-2.795 (8)	-2.795 (8)	---	---	---
Cell Death and Survival	cell death	cell death of lymphocytes	-2.384 (8)	-2.384 (8)	-2.384 (8)	---	---	---
Humoral Immune Response and Protein Synthesis	quantity	quantity of immunoglobulin	-2.086 (5)	-2.086 (5)	-2.086 (5)	---	---	---
Hematological System Development and Function, Cellular Movement, and Immune Cell Trafficking	cell movement	cell movement of leukocytes	2.161 (12)	---	---	---	---	---
Cellular Movement	migration	migration of cells	2.070 (15)	---	---	---	---	---
Cell Death and Survival	apoptosis	apoptosis	---	---	---	-2.149 (9)	-2.315 (8)	---
Cell Death and Survival	apoptosis	apoptosis of leukocytes	---	---	---	-2.218 (5)	---	---

Supplemental Table 4. Biofunctions predicted to be activated or inhibited in the spleen of non-tumor male C57BL/6 mice with different age comparisons (Adolescent (A), Young Adult (Y), Middle-Aged (M), and Old (O)) obtained through the use of Ingenuity Pathway Analysis (IPA) software. The predicted activation state of each of these functions is predicted to be increased (z-score ≥ 2) or decrease (z-score ≤ -2) by the use of the regulation z-score which indicates the degree of biofunction activity. The values in parenthesis indicate the number of genes IPA used in the prediction.

IPA Biofunctions Category	Function	Functions Annotation	Activation Z-Score (# of Genes)					
			O vs M	O vs Y	O vs A	M vs Y	M vs A	Y vs A
Cell Death and Survival	apoptosis	apoptosis of bone cancer cell lines	-2.392 (17)	---	-2.630 (8)	2.183 (16)	---	---
Cell Death and Survival	cell death	cell death of bone cancer cell lines	-2.004 (20)	---	-3.001 (13)	---	---	---
Cardiovascular Disease; Organismal Injury and Abnormalities; Tissue Morphology	size	size of infarct	---	-2.183 (6)	-2.352 (12)	---	---	-2.219 (6)
Cell Death and Survival	apoptosis	apoptosis of liver	---	-2.200 (6)	-2.183 (10)	---	---	---
Drug Metabolism; Molecular Transport; Small Molecule Biochemistry	concentration	concentration of glutathione	---	2.000 (4)	2.000 (6)	---	---	---
Cellular Development	differentiation	differentiation of cells	---	2.536 (44)	2.029 (74)	---	---	---
Cell Signaling	I-kappaB kinase/NF-kappaB cascade	I-kappaB kinase/NF-kappaB cascade	---	2.425 (6)	2.621 (9)	---	---	---
Free Radical Scavenging	metabolism	metabolism of reactive oxygen species	---	2.155 (14)	2.239 (30)	---	---	---
Cellular Movement	migration	migration of cells	---	3.537 (42)	2.027 (73)	---	---	---
Cell Death and Survival	necrosis	necrosis of liver	---	-2.412 (9)	-2.322 (16)	---	---	---
Infectious Disease	sepsis	sepsis	---	-2.449 (8)	-2.449 (9)	---	---	---
Infectious Disease	activation	activation of virus	-2.433 (7)	---	---	2.433 (7)	2.433 (7)	---
Cell Death and Survival	apoptosis	apoptosis of B lymphocytes	2.880 (16)	---	---	-2.319 (19)	-2.447 (15)	---
Cell Death and Survival	apoptosis	apoptosis of lymphocytes	2.791 (36)	---	---	-2.223 (43)	-3.040 (37)	---
Cell Death and Survival	apoptosis	apoptosis of mononuclear leukocytes	2.610 (37)	---	---	-2.076 (44)	-2.876 (38)	---
Cell Morphology; Cellular Function and Maintenance	autophagy	autophagy of cervical cancer cell lines	-2.227 (7)	---	---	2.227 (7)	2.526 (7)	---
Cell Morphology; Cellular Function and Maintenance	autophagy	autophagy of tumor cell lines	-2.838 (17)	---	---	2.607 (18)	2.132 (19)	---
Cell Morphology; Cellular Function and Maintenance	autophagy	autophagy	-3.152 (29)	---	---	2.644 (33)	2.352 (34)	---

Cell Death and Survival	cell death	cell death of lymphocytes	2.920 (41)	---	---	-2.63 (48)	-3.262 (43)	---
Cell Death and Survival	cell viability	cell viability	-2.064 (123)	---	---	2.677 (115)	2.759 (115)	---
Cell Death and Survival	necrosis	necrosis	2.181 (235)	---	---	-2.039 (227)	-2.631 (228)	---
Hematological System Development and Function; Tissue Morphology	quantity	quantity of lymphocytes	-2.692 (81)	---	---	3.233 (77)	3.233 (71)	---
Humoral Immune Response; Protein Synthesis	quantity	quantity of IgG3	-3.525 (13)	---	---	2.822 (12)	3.124 (10)	---
Embryonic Development	size	size of embryo	-3.556 (39)	---	---	3.796 (38)	3.352 (34)	---
Cell Death and Survival	survival	cell survival	-2.111 (130)	---	---	2.738 (125)	2.59 (122)	---
Gene Expression	transcription	transcription	-2.179 (163)	---	---	2.674 (164)	2.007 (158)	---
Cell Death and Survival	apoptosis	apoptosis of microglia	-2.000 (4)	---	---	2.000 (4)	---	---
Cell Morphology; Cellular Function and Maintenance	autophagy	autophagy of cells	-2.845 (24)	---	---	2.208 (27)	---	---
Cell Death and Survival	cell death	cell death of leukocyte cell lines	2.777 (22)	---	---	-2.272 (22)	---	---
Gene Expression	expression	expression of RNA	-2.060 (190)	---	---	2.757 (187)	---	---
Cellular Development; Cellular Growth and Proliferation	proliferation	proliferation of myeloma cell lines	-2.216 (7)	---	---	2.216 (7)	---	---
Hematological System Development and Function; Tissue Morphology	quantity	quantity of leukocytes	-2.470 (94)	---	---	2.935 (89)	---	---
Hematological System Development and Function; Humoral Immune Response; Tissue Morphology	quantity	quantity of B lymphocytes	-2.514 (39)	---	---	2.926 (39)	---	---
Hematological System Development and Function; Tissue Morphology	quantity	quantity of blood cells	-2.716 (103)	---	---	2.792 (97)	---	---
Gene Expression	transcription	transcription of RNA	-2.549 (155)	---	---	2.814 (159)	---	---
Cell Death and Survival	cell death	cell death of mononuclear leukocytes	2.763 (42)	---	---	---	-3.229 (44)	---
Hematological System Development and Function; Humoral Immune Response; Lymphoid Tissue Structure and Development; Tissue Morphology	quantity	quantity of follicular B lymphocytes	-2.288 (14)	---	---	---	2.059 (14)	---
Antimicrobial Response;	antimicrobial response	antimicrobial response	-2.183 (21)	---	---	---	---	---

Inflammatory Response								
Antimicrobial Response; Inflammatory Response	antiviral response	antiviral response	-2.183 (17)	---	---	---	---	---
Cell Death and Survival	cell death	cell death of hematopoietic cell lines	2.578 (25)	---	---	---	---	---
Drug Metabolism	conjugation	conjugation of glutathione	2.214 (5)	---	---	---	---	---
Cellular Development; Hematological System Development and Function; Hematopoiesis	maturation	maturation of B lymphocytes	-2.005 (10)	---	---	---	---	---
Hematological System Development and Function; Tissue Morphology	quantity	quantity of mononuclear leukocytes	-2.595 (82)	---	---	---	---	---
Cellular Movement	cell movement	cell movement	---	3.456 (46)	---	---	---	---
Cellular Movement; Hematological System Development and Function; Immune Cell Trafficking	cell movement	cell movement of leukocytes	---	2.608 (23)	---	---	---	---
Cellular Movement; Hematological System Development and Function; Immune Cell Trafficking	cell movement	cell movement of mononuclear leukocytes	---	2.431 (14)	---	---	---	---
Cellular Movement	chemotaxis	chemotaxis of cells	---	2.558 (14)	---	---	---	---
Cellular Movement; Hematological System Development and Function; Immune Cell Trafficking; Inflammatory Response	chemotaxis	chemotaxis of mononuclear leukocytes	---	2.203 (9)	---	---	---	---
Cellular Movement; Hematological System Development and Function; Immune Cell Trafficking; Inflammatory Response	chemotaxis	chemotaxis of leukocytes	---	2.173 (12)	---	---	---	---
Cardiovascular Disease; Organismal Injury and Abnormalities	damage	damage of heart	---	-2.219 (5)	---	---	---	---
Cellular Development; Hematological System Development and Function;	development	development of blood cells	---	2.120 (19)	---	---	---	---

Hematopoiesis								
Cellular Development; Hematological System Development and Function; Hematopoiesis; Lymphoid Tissue Structure and Development	development	development of leukocytes	---	2.120 (16)	---	---	---	---
Embryonic Development; Lymphoid Tissue Structure and Development; Organ Development; Organismal Development	development	development of lymphatic system component	---	2.224 (12)	---	---	---	---
Cellular Development; Hematopoiesis	differentiation	differentiation of bone marrow cells	---	2.219 (7)	---	---	---	---
Organismal Injury and Abnormalities	fibrosis	Fibrosis	---	-2.270 (14)	---	---	---	---
Inflammatory Response; Respiratory Disease	inflammation	inflammation of lung	---	-2.359 (14)	---	---	---	---
Inflammatory Response	inflammation	inflammation of organ	---	-2.880 (29)	---	---	---	---
Cellular Movement; Immune Cell Trafficking	migration	leukocyte migration	---	3.044 (27)	---	---	---	---
Cellular Movement	migration	migration of tumor cell lines	---	2.398 (19)	---	---	---	---
Cellular Movement; Nervous System Development and Function	migration	migration of neurons	---	2.370 (9)	---	---	---	---
Cellular Movement; Embryonic Development	migration	migration of embryonic cells	---	2.156 (5)	---	---	---	---
Cellular Assembly and Organization; Cellular Function and Maintenance	organization	organization of cytoskeleton	---	2.228 (26)	---	---	---	---
Cell-To-Cell Signaling and Interaction; Cellular Function and Maintenance; Inflammatory Response	phagocytosis	phagocytosis of cells	---	2.279 (8)	---	---	---	---
Cellular Function and Maintenance; Inflammatory Response	phagocytosis	phagocytosis	---	2.488 (9)	---	---	---	---
Cell Morphology; Cellular Assembly and Organization; Cellular Function and Maintenance	reorganization	reorganization of cytoskeleton	---	2.219 (6)	---	---	---	---
Free Radical Scavenging	synthesis	synthesis of reactive oxygen	---	2.001 (13)	---	---	---	---

		species						
Cell Death and Survival; Nervous System Development and Function	cell viability	cell viability of neurons	---	---	2.222 (12)	---	---	2.186 (7)
Cardiovascular Disease	infarction	Infarction	---	---	-2.556 (15)	---	---	-2.219 (8)
Cell Death and Survival	apoptosis	apoptosis of granule cells	---	---	-2.000 (5)	---	---	---
Cell Death and Survival	apoptosis	apoptosis of liver	---	---	-2.375 (11)	---	---	---
Cell-To-Cell Signaling and Interaction	binding	binding of cells	---	---	2.142 (25)	---	---	---
Cancer; Immunological Disease	hyperplasia	hyperplasia of lymphoid organ	---	---	2.607 (7)	---	---	---
Developmental Disorder	hypertrophy	hypertrophy of cells	---	---	2.984 (14)	---	---	---
Cellular Movement; Hematological System Development and Function; Immune Cell Trafficking; Inflammatory Response	migration	migration of macrophages	---	---	2.416 (6)	---	---	---
Free Radical Scavenging	production	production of reactive oxygen species	---	---	2.042 (22)	---	---	---
Nutritional Disease	weight loss	weight loss	---	---	-2.051 (12)	---	---	---
Cell Death and Survival	apoptosis	apoptosis of pheochromocytoma cell lines	---	---	---	2.601 (13)	2.312 (13)	---
Cell Death and Survival	cell death	cell death of hematopoietic cell lines	---	---	---	-2.101 (22)	-2.267 (21)	---
Cell Death and Survival	cell death	cell death	---	---	---	-2.155 (287)	-2.731 (281)	---
Cell Death and Survival	cell death	cell death of lymphoid organ	---	---	---	-2.357 (19)	-2.890 (17)	---
Hematological System Development and Function; Humoral Immune Response; Tissue Morphology	quantity	quantity of B lymphocytes	---	---	---	2.926 (39)	2.929 (33)	---
Hematological System Development and Function; Tissue Morphology	quantity	quantity of leukocytes	---	---	---	2.935 (89)	2.905 (84)	---
Hematological System Development and Function; Tissue Morphology	quantity	quantity of blood cells	---	---	---	2.792 (97)	2.585 (92)	---
Cell Cycle; Cellular Development; Connective Tissue Development and Function	senescence	senescence of fibroblast cell lines	---	---	---	2.183 (11)	2.155 (11)	---

Cell Morphology; Connective Tissue Development and Function	size	size of adipocytes	---	---	---	2.111 (11)	2.111 (11)	---
Cellular Assembly and Organization	aggregation	aggregation of membrane rafts	---	---	---	-2.000 (4)	---	---
Hematological Disease	blood protein disorder	blood protein disorder	---	---	---	-2.000 (33)	---	---
Cell Death and Survival	cell death	cell death of pheochromocytoma cell lines	---	---	---	2.368 (15)	---	---
Cell Death and Survival	cell death	cell death of lymphocytes	---	---	---	-2.661 (47)	---	---
Cellular Movement	cell movement	cell movement of tumor cell lines	---	---	---	3.100 (63)	---	---
Cell Death and Survival	cell viability	cell viability of melanoma cell lines	---	---	---	2.219 (6)	---	---
DNA Replication, Recombination, and Repair	damage	DNA damage	---	---	---	2.010 (19)	---	---
Gastrointestinal Disease; Hepatic System Disease; Organismal Injury and Abnormalities	degeneration	degeneration of liver	---	---	---	-2.415 (6)	---	---
Organismal Injury and Abnormalities	degeneration	Organ Degeneration	---	---	---	-3.243 (18)	---	---
Cellular Development; Hematological System Development and Function; Hematopoiesis	maturation	maturation of lymphocytes	---	---	---	2.045 (15)	---	---
Cellular Development; Hematopoiesis	maturation	maturation of leukocytes	---	---	---	2.189 (23)	---	---
Cellular Development	maturation	maturation of blood cells	---	---	---	2.294 (24)	---	---
Neurological Disease; Organismal Injury and Abnormalities	neurodegeneration	neurodegeneration of cerebellum	---	---	---	-2.000 (5)	---	---
Neurological Disease; Skeletal and Muscular Disorders	neuromuscular disease	neuromuscular disease	---	---	---	2.205 (80)	---	---
Amino Acid Metabolism; Post-Translational Modification; Small Molecule Biochemistry	phosphorylation	phosphorylation of L-serine	---	---	---	2.415 (6)	---	---
Cancer	transformation	cell transformation	---	---	---	2.106 (44)	---	---
Cancer	transformation	transformation of fibroblast cell lines	---	---	---	2.457 (27)	---	---
Cell Death and Survival	apoptosis	apoptosis	---	---	---	---	-2.034 (232)	---
Cell Death and Survival	apoptosis	apoptosis of T lymphocytes	---	---	---	---	-2.124 (29)	---
Cell Death and Survival	apoptosis	apoptosis of prostate cancer cell lines	---	---	---	---	-2.358 (18)	---
Cell Death and Survival	apoptosis	apoptosis of leukocytes	---	---	---	---	-2.675 (47)	---

Cell Death and Survival	cell death	cell death of prostate cancer cell lines	---	---	---	---	-2.091 (20)	---
Cell Death and Survival	cell death	cell death of tumor cell lines	---	---	---	---	-2.185 (147)	---
Cell Death and Survival	cell death	cell death of immune cells	---	---	---	---	-2.208 (67)	---
Cell Death and Survival	cell death	cell death of thymocytes	---	---	---	---	-2.628 (15)	---
Cell Death and Survival	cell death	cell death of T lymphocytes	---	---	---	---	-2.785 (35)	---
Cell Death and Survival; Nervous System Development and Function	cell viability	cell viability of cortical neurons	---	---	---	---	2.425 (8)	---
Protein Degradation; Protein Synthesis	degradation	degradation of protein	---	---	---	---	2.023 (47)	---
Protein Synthesis	expression	expression of protein	---	---	---	---	-2.539 (42)	---
Cellular Growth and Proliferation; Connective Tissue Development and Function; Skeletal and Muscular System Development and Function	formation	formation of osteoclasts	---	---	---	---	2.049 (11)	---
Cell Signaling	protein kinase cascade	protein kinase cascade	---	---	---	---	2.365 (39)	---
Hematological System Development and Function; Lymphoid Tissue Structure and Development	quantity	quantity of follicular B lymphocytes	---	---	---	---	2.059 (14)	---
Cell Cycle	senescence	senescence of cells	---	---	---	---	2.729 (22)	---
Cellular Compromise	stress response	stress response of cells	---	---	---	---	-2.588 (12)	---
Protein Synthesis	translation	translation	---	---	---	---	-2.714 (39)	---
Protein Synthesis	translation	translation of protein	---	---	---	---	-2.714 (38)	---
Cell-To-Cell Signaling and Interaction; Hematological System Development and Function; Immune Cell Trafficking; Inflammatory Response	activation	activation of leukocytes	---	---	---	---	---	-2.179 (14)
Cell-To-Cell Signaling and Interaction; Hematological System Development and Function; Immune Cell Trafficking; Inflammatory Response	activation	activation of lymphocytes	---	---	---	---	---	-2.392 (10)

Cell Death and Survival	cell death	cell death of lung cancer cell lines	---	---	---	---	---	-2.556 (8)
Cellular Development; Hematopoiesis	maturation	maturation of leukocytes	---	---	---	---	---	-2.197 (7)
Cellular Movement	migration	migration of breast cancer cell lines	---	---	---	---	---	2.387 (6)
Cellular Development; Cellular Growth and Proliferation; Hematological System Development and Function	proliferation	proliferation of T lymphocytes	---	---	---	---	---	-2.360 (16)
Cellular Development; Cellular Growth and Proliferation; Hematological System Development and Function	proliferation	proliferation of immune cells	---	---	---	---	---	-3.045 (21)
Cellular Development; Cellular Growth and Proliferation; Hematological System Development and Function; Humoral Immune Response	proliferation	proliferation of B lymphocytes	---	---	---	---	---	-3.108 (10)
Cellular Development; Cellular Growth and Proliferation; Hematological System Development and Function	proliferation	proliferation of lymphocytes	---	---	---	---	---	-3.271 (20)
Humoral Immune Response; Protein Synthesis	quantity	quantity of IgG	---	---	---	---	---	-2.168 (10)
Humoral Immune Response; Protein Synthesis	quantity	quantity of immunoglobulin	---	---	---	---	---	-2.336 (13)
Infectious Disease	replication	replication of Influenza A virus	---	---	---	---	---	-2.813 (9)
Organismal Injury and Abnormalities; Tissue Morphology	size	size of lesion	---	---	---	---	---	-2.078 (9)

Supplemental Table 5. Upstream regulators predicted to be activated or inhibited (as indicated by the activation z-score) in the spleen of LLC tumor bearing C57BL/6 male mice with different age comparisons (Adolescent (A), Young Adult (Y), Middle-Aged (M), and Old (O)) obtained through the use of Ingenuity Pathway Analysis (IPA) software. Regulation z-score indicates the degree of inhibition (for negative values) or activation (for positive values). The third column denotes the effects the upstream regulators have on tumor progression based on the literature (reference in parenthesis).

Upstream Regulator	Molecule Type	Effects on Tumor (Ref.)	Activation Z-score (# of Genes)					
			O vs M	O vs Y	O vs A	MA vs Y	M vs A	Y vs A
Tnf (family)	group	Both [1]	2.000 (4)	2.219 (5)	2.219 (5)	2.000 (4)	2.000 (4)	---
CHUK	kinase	Promotes [2]	2.158 (6)	2.158 (6)	2.158 (6)	---	---	---
IFNG	cytokine	Inhibits [3]	2.879 (14)	2.879 (15)	2.879 (15)	---	---	---
IKBKB	kinase	Promotes [4]	2.178 (5)	2.178 (5)	2.178 (5)	---	---	---
IRF3	transcription regulator	Inhibits [5]	2.169 (6)	2.169 (6)	2.169 (6)	---	---	---
IRF7	transcription regulator	Inhibits [5]	2.585 (7)	2.585 (7)	2.585 (7)	---	---	---
NFκB (complex)	complex	Promotes [6]	2.289 (9)	2.080 (8)	2.289 (9)	---	---	---
NFκBIA	transcription regulator	Inhibits [7]	2.289 (7)	2.061 (6)	2.289 (7)	---	---	---
TNF	cytokine	Both [1]	2.561 (13)	2.149 (13)	2.353 (14)	---	---	---
STAT3	transcription regulator	Promotes [8]	2.431 (6)	2.431 (6)	2.431 (6)	---	2.217 (5)	---
mir-21	microRNA	Promotes [9]	-2.000 (4)	-2.000 (4)	-2.000 (4)	---	---	---
IFNA2	cytokine	Inhibits [10]	---	2.193 (5)	2.193 (5)	---	---	---
Interferon alpha	group	Inhibits [10]	---	2.010 (8)	2.010 (8)	---	---	---
RELA	transcription regulator	Promotes [11]	2.149 (9)	---	2.149 (9)	---	---	---
NKX2-3	transcription regulator	Promotes [12]	-2.000 (4)	---	---	---	---	---
TGFβ1	growth factor	Promotes [13]	---	2.029 (10)	---	---	---	---

1. Mocellin S and Nitti D. TNF and cancer: the two sides of the coin. Front Biosci. 2008; 13:2774-2783.
2. Alameda JP, Moreno-Maldonado R, Fernandez-Acenero MJ, Navarro M, Page A, Jorcano JL, Bravo A, Ramirez A and Casanova ML. Increased IKKalpha expression in the basal layer of the epidermis of transgenic mice enhances the malignant potential of skin tumors. PLoS One. 2011; 6(7):e21984.

3. Lewis DA, Travers JB and Spandau DF. A new paradigm for the role of aging in the development of skin cancer. J Invest Dermatol. 2009; 129(3):787-791.
4. Chariot A. The NF-kappaB-independent functions of IKK subunits in immunity and cancer. Trends Cell Biol. 2009; 19(8):404-413.
5. Romieu-Mourez R, Solis M, Nardin A, Goubau D, Baron-Bodo V, Lin R, Massie B, Salcedo M and Hiscott J. Distinct roles for IFN regulatory factor (IRF)-3 and IRF-7 in the activation of antitumor properties of human macrophages. Cancer Res. 2006; 66(21):10576-10585.
6. Alberti C, Pinciroli P, Valeri B, Ferri R, Ditto A, Umezawa K, Sensi M, Canevari S and Tomassetti A. Ligand-dependent EGFR activation induces the co-expression of IL-6 and PAI-1 via the NFkB pathway in advanced-stage epithelial ovarian cancer. Oncogene. 2012; 31(37):4139-4149.
7. Bredel M, Scholtens DM, Yadav AK, Alvarez AA, Renfrow JJ, Chandler JP, Yu IL, Carro MS, Dai F, Tagge MJ, Ferrarese R, Bredel C, Phillips HS, Lukac PJ, Robe PA, Weyerbrock A, et al. NFKBIA deletion in glioblastomas. N Engl J Med. 2011; 364(7):627-637.
8. Wu J, Patmore DM, Jousma E, Eaves DW, Breving K, Patel AV, Schwartz EB, Fuchs JR, Cripe TP, Stemmer-Rachamimov AO and Ratner N. EGFR-STAT3 signaling promotes formation of malignant peripheral nerve sheath tumors. Oncogene. 2013.
9. Sicard F, Gayral M, Lulka H, Buscail L and Cordelier P. Targeting miR-21 for the therapy of pancreatic cancer. Mol Ther. 2013; 21(5):986-994.
10. Nakaji M, Yano Y, Ninomiya T, Seo Y, Hamano K, Yoon S, Kasuga M, Teramoto T, Hayashi Y and Yokozaki H. IFN-alpha prevents the growth of pre-neoplastic lesions and inhibits the development of hepatocellular carcinoma in the rat. Carcinogenesis. 2004; 25(3):389-397.
11. Lu X, An H, Jin R, Zou M, Guo Y, Su PF, Liu D, Shyr Y and Yarbrough WG. PPM1A is a RelA phosphatase with tumor suppressor-like activity. Oncogene. 2013.
12. Yu W, Lin Z, Pastor DM, Hegarty JP, Chen X, Kelly AA, Wang Y, Poritz LS and Koltun WA. Genes regulated by Nkx2-3 in sporadic and inflammatory bowel disease-associated colorectal cancer cell lines. Dig Dis Sci. 2010; 55(11):3171-3180.
13. Nguyen DH, Martinez-Ruiz H and Barcellos-Hoff MH. Consequences of epithelial or stromal TGFbeta1 depletion in the mammary gland. J Mammary Gland Biol Neoplasia. 2011; 16(2):147-155.

Supplemental Table 6. Upstream regulators predicted to be activated or inhibited (as indicated by the activation z-score) in the spleen of non-tumor bearing C57BL/6 male mice with different age comparisons (Adolescent (A), Young Adult (Y), Middle-Aged (M), and Old (O)) obtained through the use of Ingenuity Pathway Analysis (IPA) software. Regulation z-score indicates the degree of inhibition (for negative values) or activation (for positive values). The third column denotes the effects the upstream regulators have on tumor progression based on the literature (reference in parenthesis).

Upstream Regulator	Molecule Type	Effects on Tumor (Ref.)	Activation Z-Score (# of Genes)					
			O vs MA	O vs Y	O vs A	MA vs Y	MA vs A	Y vs A
MYCN	transcription regulator	Promotes [1]	2.985 (39)	-2.425 (6)	-2.728 (11)	-4.079 (40)	-3.908 (42)	---
IL6	cytokine	Promotes [2]	-2.323 (35)	2.157 (17)	---	2.61 (34)	2.605 (32)	---
NR3C2	ligand-dependent nuclear receptor	Promotes [3]	2.236 (9)	---	2.000 (5)	-2.449 (8)	---	---
PPARG	ligand-dependent nuclear receptor	Inhibits [4]	2.615 (17)	---	2.005 (10)	---	---	---
MYC	transcription regulator	Promotes [5]	---	-2.947 (18)	-2.317 (23)	-2.354 (75)	---	---
STAT6	transcription regulator	Promotes [6]	---	2.000 (5)	2.795 (10)	---	2.449 (10)	2.414 (3.2)
KRAS	enzyme	Promotes [7]	---	-2.178 (9)	-2.201 (14)	---	-2.122 (30)	---
IKBKG	kinase	Promotes [8]	---	2.183 (5)	2.183 (5)	---	---	---
CD24	other	Promotes [9]	-2.635 (10)	---	---	2.438 (9)	2.000 (7)	---
CSF2	cytokine	Promotes [10]	-2.552 (27)	---	---	3.125 (31)	2.603 (30)	---
CTNNB1	transcription regulator	Promotes [11]	-2.412 (24)	---	---	2.789 (26)	2.607 (27)	---
EIF4E	translation regulator	Promotes [12]	3.004 (18)	---	---	-3.748 (20)	-3.154 (19)	---
FOXO1	transcription regulator	Inhibits [13]	2.516 (25)	---	---	-2.283 (24)	-2.559 (22)	---
HGF	growth factor	Promotes [14]	-2.552 (22)	---	---	2.584 (23)	2.415 (22)	---
HSF2	transcription regulator	Promotes [15]	-2.000 (5)	---	---	2.000 (5)	2.000 (5)	---
Map4k4	kinase	Promotes [16]	-2.496 (13)	---	---	2.111 (11)	2.309 (12)	---
NFATC2	transcription regulator	Promotes [17]	-2.189 (8)	---	---	2.189 (9)	2.189 (9)	---
OSM	cytokine	Promotes [18]	-2.281 (26)	---	---	2.514 (25)	2.471 (25)	---
ANXA2	other	Promotes [19]	2.000 (4)	---	---	-2.000 (4)	---	---
FADD	other	Promotes [20]	-2.000 (5)	---	---	2.236 (6)	---	---
GFI1	transcription regulator	Promotes [21]	2.598 (7)	---	---	-2.954 (9)	---	---
Ifnar	group	Inhibits [22]	-2.157 (8)	---	---	2.573 (7)	---	---

IFNK	cytokine	N.D.	-2.000 (4)	---	---	2.000 (4)	---	---
IL12 (complex)	complex	Inhibits [23]	-2.574 (11)	---	---	2.404 (8)	---	---
IL2	cytokine	Inhibits [24]	-2.034 (32)	---	---	2.314 (33)	---	---
Vegf	group	Promotes [25]	-2.200 (15)	---	---	2.917 (16)	3.513 (16)	---
INHA	growth factor	Promotes [26]	-2.000 (4)	---	---	---	---	-2.000 (4)
ANXA7	ion channel	Inhibits [27]	2.000 (4)	---	---	---	---	---
GNA12	enzyme	Promotes [28]	-2.360 (9)	---	---	---	---	---
PDX1	transcription regulator	Promotes [29]	-2.137 (10)	---	---	---	---	---
PTEN	phosphatase	Inhibits [30]	-2.445 (35)	---	---	---	---	---
RAB1B	other	Promotes [31]	2.000 (4)	---	---	---	---	---
RHO	G-protein coupled receptor	N.D.	-2.630 (7)	---	---	---	---	---
SLC13A1	transporter	Inhibits [32]	2.000 (4)	---	---	---	---	---
THRB	ligand-dependent nuclear receptor	Promotes [33]	2.425 (9)	---	---	---	---	---
TWIST1	transcription regulator	Promotes [34]	-2.000 (4)	---	---	---	---	---
AKT1	kinase	Promotes [35]	---	2.219 (5)	---	---	---	---
Alpha catenin	group	Inhibits [36]	---	-2.415 (6)	---	-2.433 (6)	-2.425 (6)	---
CXCL12	cytokine	Promotes [37]	---	2.000 (4)	---	---	2.164 (11)	---
CD44	enzyme	Both [38]	---	2.425 (9)	---	---	---	---
IGF1	growth factor	Promotes [39]	---	2.294 (15)	---	---	---	---
IL4	cytokine	Promotes [40]	---	2.026 (12)	---	---	---	---
IL5	cytokine	Promotes [41]	---	2.377 (7)	---	---	---	---
PLAG1	transcription regulator	Promotes [42]	---	---	2.219 (5)	---	2.236 (5)	2.000 (4)
ESRRA	ligand-dependent nuclear receptor	Promotes [43]	---	---	2.219 (5)	---	---	2.630 (7)
CEBPA	transcription regulator	Inhibits [44]	---	---	2.137 (12)	---	---	---
CHUK	kinase	Promotes [45]	---	---	2.414 (6)	---	---	---
CSF1	cytokine	Promotes [46]	---	---	2.177 (7)	---	---	---
HNF4A	transcription regulator	Inhibits [47]	---	---	2.000 (34)	---	---	---
KITLG	growth factor	Promotes [48]	---	---	2.400 (8)	---	---	---
MAPK1	kinase	Promotes [49]	---	---	-2.138 (9)	---	---	---
PSEN2	peptidase	N.D.	---	---	-2.213 (5)	---	---	---
STAT5A	transcription regulator	Promotes [50]	---	---	2.219 (5)	---	---	---
STAT5B	transcription regulator	Promotes [51]	---	---	2.005 (9)	---	---	---

TGFB1	growth factor	Both [52]	---	---	2.527 (45)	---	---	---
TNFRSF1B	transmembrane receptor	Promotes [53]	---	---	-2.000 (4)	---	---	---
CTGF	growth factor	Promotes [54]	---	---	---	2.000 (4)	2.000 (4)	---
ERK	group	Promotes [55]	---	---	---	2.138 (9)	2.138 (9)	---
IL3	cytokine	Promotes [56]	---	---	---	2.000 (5)	2.000 (5)	---
INSR	kinase	Promotes [57]	---	---	---	-2.282 (39)	-2.282 (39)	---
MITF	transcription regulator	Promotes [58]	---	---	---	2.236 (7)	2.449 (7)	---
PDGF BB	complex	Promotes [59]	---	---	---	2.000 (17)	2.279 (16)	---
TNF	cytokine	Both [60]	---	---	---	2.194 (78)	---	-2.563 (23)
ERG	transcription regulator	Promotes [61]	---	---	---	2.53 (12)	---	---
FGF2	growth factor	Promotes [62]	---	---	---	2.216 (14)	---	---
IFN Beta	group	Inhibits [63]	---	---	---	2.200 (7)	---	---
IRF1	transcription regulator	Inhibits [64]	---	---	---	2.569 (8)	---	---
IRF7	transcription regulator	Inhibits [65]	---	---	---	2.213 (11)	---	---
IRF8	transcription regulator	Inhibits [66]	---	---	---	2.566 (8)	---	---
KDM5B	transcription regulator	Promotes [67]	---	---	---	-2.121 (8)	---	---
P38 MAPK	group	Inhibits [68]	---	---	---	2.205 (17)	---	---
PPARGC1A	transcription regulator	Promotes [69]	---	---	---	-2.008 (12)	---	---
SOCS1	other	Inhibits [70]	---	---	---	-2.203 (6)	---	---
STAT1	transcription regulator	Promotes [71]	---	---	---	2.174 (18)	---	---
Tlr	group	Both [72]	---	---	---	2.172 (5)	---	---
TLR4	transmembrane receptor	Both [73]	---	---	---	2.401 (17)	---	---
VIP	other	Inhibits [74]	---	---	---	-2.197 (7)	---	---
Cg	complex	Promotes [75]	---	---	---	---	2.361 (11)	---
NEUROG1	transcription regulator	N.D.	---	---	---	---	-2.000 (4)	---
PDGF (family)	group	Promotes [25]	---	---	---	---	2.000 (4)	---
Retnlb	other	Inhibits [76]	---	---	---	---	2.000 (4)	---
SPI1	transcription regulator	Inhibits [77]	---	---	---	---	2.412 (11)	---
CD40LG	cytokine	Promotes [78]	---	---	---	---	---	-2.602 (11)
IFNG	cytokine	Inhibits [79]	---	---	---	---	---	-2.540 (23)
Interferon alpha	group	Inhibits [80]	---	---	---	---	---	-2.575 (9)

1. Calao M, Sekyere EO, Cui HJ, Cheung BB, Thomas WD, Keating J, Chen JB, Raif A, Jankowski K, Davies NP, Bekkum MV, Chen B, Tan O, Ellis T, Norris MD, Haber M, et al. Direct effects of Bmi1 on p53 protein stability inactivates oncoprotein stress responses in embryonal cancer precursor cells at tumor initiation. Oncogene. 2013; 32(31):3616-3626.
2. Korkaya H, Kim GI, Davis A, Malik F, Henry NL, Ithimakin S, Quraishi AA, Tawakkol N, D'Angelo R, Paulson AK, Chung S, Luther T, Paholak HJ, Liu S, Hassan KA, Zen Q, et al. Activation of an IL6 inflammatory loop mediates trastuzumab resistance in HER2+ breast cancer by expanding the cancer stem cell population. Mol Cell. 2012; 47(4):570-584.
3. Krauthammer M, Kong Y, Ha BH, Evans P, Bacchiocchi A, McCusker JP, Cheng E, Davis MJ, Goh G, Choi M, Ariyan S, Narayan D, Dutton-Regester K, Capatana A, Holman EC, Bosenberg M, et al. Exome sequencing identifies recurrent somatic RAC1 mutations in melanoma. Nat Genet. 2012; 44(9):1006-1014.
4. Koeffler HP. Peroxisome proliferator-activated receptor gamma and cancers. Clin Cancer Res. 2003; 9(1):1-9.
5. Miller DM, Thomas SD, Islam A, Muench D and Sedoris K. c-Myc and cancer metabolism. Clin Cancer Res. 2012; 18(20):5546-5553.
6. Merk BC, Owens JL, Lopes MB, Silva CM and Hussaini IM. STAT6 expression in glioblastoma promotes invasive growth. BMC Cancer. 2011; 11:184.
7. Huang H, Daniluk J, Liu Y, Chu J, Li Z, Ji B and Logsdon CD. Oncogenic K-Ras requires activation for enhanced activity. Oncogene. 2013.
8. Wolf MJ, Seleznik GM, Zeller N and Heikenwalder M. The unexpected role of lymphotoxin beta receptor signaling in carcinogenesis: from lymphoid tissue formation to liver and prostate cancer development. Oncogene. 2010; 29(36):5006-5018.
9. Overdevest JB, Knubel KH, Duex JE, Thomas S, Nitz MD, Harding MA, Smith SC, Frierson HF, Conaway M and Theodorescu D. CD24 expression is important in male urothelial tumorigenesis and metastasis in mice and is androgen regulated. Proc Natl Acad Sci U S A. 2012; 109(51):E3588-3596.
10. Uemura Y, Kobayashi M, Nakata H, Kubota T, Bandobashi K, Saito T and Taguchi H. Effects of GM-CSF and M-CSF on tumor progression of lung cancer: roles of MEK1/ERK and AKT/PKB pathways. Int J Mol Med. 2006; 18(2):365-373.
11. Wend P, Runke S, Wend K, Anchondo B, Yesayan M, Jardon M, Hardie N, Loddenkemper C, Ulasov I, Lesniak MS, Wolsky R, Bentolila LA, Grant SG, Elashoff D, Lehr S, Latimer JJ, et al. WNT10B/beta-catenin signalling induces HMGA2 and proliferation in metastatic triple-negative breast cancer. EMBO Mol Med. 2013.
12. Wu M, Liu Y, Di X, Kang H, Zeng H, Zhao Y, Cai K, Pang T, Wang S, Yao Y and Hu X. EIF4E over-expresses and enhances cell proliferation and cell cycle progression in nasopharyngeal carcinoma. Med Oncol. 2013; 30(1):400.
13. Zhang H, Pan Y, Zheng L, Choe C, Lindgren B, Jensen ED, Westendorf JJ, Cheng L and Huang H. FOXO1 inhibits Runx2 transcriptional activity and prostate cancer cell migration and invasion. Cancer Res. 2011; 71(9):3257-3267.
14. Cecchi F, Rabe DC and Bottaro DP. Targeting the HGF/Met signaling pathway in cancer therapy. Expert Opin Ther Targets. 2012; 16(6):553-572.
15. De Thonel A, Mezger V and Garrido C. Implication of heat shock factors in tumorigenesis: therapeutical potential. Cancers (Basel). 2011; 3(1):1158-1181.
16. Qiu MH, Qian YM, Zhao XL, Wang SM, Feng XJ, Chen XF and Zhang SH. Expression and prognostic significance of MAP4K4 in lung adenocarcinoma. Pathol Res Pract. 2012; 208(9):541-548.
17. Gerlach K, Daniel C, Lehr HA, Nikolaev A, Gerlach T, Atreya R, Rose-John S, Neurath MF and Weigmann B. Transcription factor NFATc2 controls the emergence of colon cancer associated with IL-6-dependent colitis. Cancer Res. 2012; 72(17):4340-4350.
18. Bolin C, Tawara K, Sutherland C, Redshaw J, Aranda P, Moselhy J, Anderson R and Jorcyk CL. Oncostatin m promotes mammary tumor metastasis to bone and osteolytic bone degradation. Genes Cancer. 2012; 3(2):117-130.
19. Madureira PA, Hill R, Miller VA, Giacomantonio C, Lee PW and Waisman DM. Annexin A2 is a novel cellular redox regulatory protein involved in tumorigenesis. Oncotarget. 2011; 2(12):1075-1093.
20. Tourneur L, Buzyn A and Chiocchia G. FADD adaptor in cancer. Med Immunol. 2005; 4(1):1.
21. Khandanpour C, Phelan JD, Vassen L, Schutte J, Chen R, Horman SR, Gaudreau MC, Krongold J, Zhu J, Paul WE, Duhrsen U, Gottgens B, Grimes HL and Moroy T. Growth factor independence 1 antagonizes a p53-induced DNA damage response pathway in lymphoblastic leukemia. Cancer Cell. 2013; 23(2):200-214.
22. Bhattacharya S, HuangFu WC, Dong G, Qian J, Baker DP, Karar J, Koumenis C, Diehl JA and Fuchs SY. Anti-tumorigenic effects of Type 1 interferon are subdued by integrated stress responses. Oncogene. 2013; 32(36):4214-4221.

23. Murdoch C, Muthana M, Coffelt SB and Lewis CE. The role of myeloid cells in the promotion of tumour angiogenesis. Nat Rev Cancer. 2008; 8(8):618-631.
24. Antony GK and Dudek AZ. Interleukin 2 in cancer therapy. Curr Med Chem. 2010; 17(29):3297-3302.
25. Peterson JE, Zurakowski D, Italiano JE, Jr., Michel LV, Connors S, Oenick M, D'Amato RJ, Klement GL and Folkman J. VEGF, PF4 and PDGF are elevated in platelets of colorectal cancer patients. Angiogenesis. 2012; 15(2):265-273.
26. Risbridger GP, Shibata A, Ferguson KL, Stamey TA, McNeal JE and Peehl DM. Elevated expression of inhibin alpha in prostate cancer. J Urol. 2004; 171(1):192-196.
27. Srivastava M, Torosyan Y, Raffeld M, Eidelman O, Pollard HB and Bubendorf L. ANXA7 expression represents hormone-relevant tumor suppression in different cancers. Int J Cancer. 2007; 121(12):2628-2636.
28. Ha JH, Ward JD, Varadarajalu L, Kim SG and Dhanasekaran DN. The gep proto-oncogene Galpha12 mediates LPA-stimulated activation of CREB in ovarian cancer cells. Cell Signal. 2014; 26(1):122-132.
29. Liu SH, Patel S, Gingras MC, Nemunaitis J, Zhou G, Chen C, Li M, Fisher W, Gibbs R and Brunicardi FC. PDX-1: demonstration of oncogenic properties in pancreatic cancer. Cancer. 2011; 117(4):723-733.
30. Knobbe CB, Lapin V, Suzuki A and Mak TW. The roles of PTEN in development, physiology and tumorigenesis in mouse models: a tissue-by-tissue survey. Oncogene. 2008; 27(41):5398-5415.
31. Zhai H, Song B, Xu X, Zhu W and Ju J. Inhibition of autophagy and tumor growth in colon cancer by miR-502. Oncogene. 2013; 32(12):1570-1579.
32. Dawson PA, Choyce A, Chuang C, Whitelock J, Markovich D and Leggatt GR. Enhanced tumor growth in the NaS1 sulfate transporter null mouse. Cancer Sci. 2010; 101(2):369-373.
33. Zambrano A, Garcia-Carpizo V, Gallardo ME, Villamuera R, Gomez-Ferreria MA, Pascual A, Buisine N, Sachs LM, Garesse R and Aranda A. The thyroid hormone receptor beta induces DNA damage and premature senescence. J Cell Biol. 2014; 204(1):129-146.
34. Gajula RP, Chettiar ST, Williams RD, Thiyagarajan S, Kato Y, Aziz K, Wang R, Gandhi N, Wild AT, Vesuna F, Ma J, Salih T, Cades J, Fertig E, Biswal S, Burns TF, et al. The twist box domain is required for Twist1-induced prostate cancer metastasis. Mol Cancer Res. 2013; 11(11):1387-1400.
35. Huang CY, Fong YC, Lee CY, Chen MY, Tsai HC, Hsu HC and Tang CH. CCL5 increases lung cancer migration via PI3K, Akt and NF-kappaB pathways. Biochem Pharmacol. 2009; 77(5):794-803.
36. Mantovani A, Garlanda C and Allavena P. Molecular pathways and targets in cancer-related inflammation. Ann Med. 2010; 42(3):161-170.
37. Hattermann K and Mentlein R. An infernal trio: the chemokine CXCL12 and its receptors CXCR4 and CXCR7 in tumor biology. Ann Anat. 2013; 195(2):103-110.
38. Naor D, Nedvetzki S, Golan I, Melnik L and Faitelson Y. CD44 in cancer. Crit Rev Clin Lab Sci. 2002; 39(6):527-579.
39. Tian X, Hao K, Qin C, Xie K, Xie X and Yang Y. Insulin-Like Growth Factor 1 Receptor Promotes the Growth and Chemoresistance of Pancreatic Cancer. Dig Dis Sci. 2013.
40. Li Z, Jiang J, Wang Z, Zhang J, Xiao M, Wang C, Lu Y and Qin Z. Endogenous interleukin-4 promotes tumor development by increasing tumor cell resistance to apoptosis. Cancer Res. 2008; 68(21):8687-8694.
41. Simson L, Ellyard JI, Dent LA, Matthaei KI, Rothenberg ME, Foster PS, Smyth MJ and Parish CR. Regulation of carcinogenesis by IL-5 and CCL11: a potential role for eosinophils in tumor immune surveillance. J Immunol. 2007; 178(7):4222-4229.
42. Hibbard MK, Kozakewich HP, Dal Cin P, Sciot R, Tan X, Xiao S and Fletcher JA. PLAG1 fusion oncogenes in lipoblastoma. Cancer Res. 2000; 60(17):4869-4872.
43. Deblois G, Chahrour G, Perry MC, Sylvain-Drolet G, Muller WJ and Giguere V. Transcriptional control of the ERBB2 amplicon by ERRalpha and PGC-1beta promotes mammary gland tumorigenesis. Cancer Res. 2010; 70(24):10277-10287.
44. Grossmann V, Bacher U, Kohlmann A, Butschalowski K, Roller A, Jeromin S, Dicker F, Kern W, Schnittger S, Haferlach T and Haferlach C. Expression of CEBPA is reduced in RUNX1-mutated acute myeloid leukemia. Blood Cancer J. 2012; 2:e86.
45. Alameda JP, Moreno-Maldonado R, Fernandez-Acenero MJ, Navarro M, Page A, Jorcano JL, Bravo A, Ramirez A and Casanova ML. Increased IKKalpha expression in the basal layer of the epidermis of transgenic mice enhances the malignant potential of skin tumors. PLoS One. 2011; 6(7):e21984.
46. Espinosa I, Edris B, Lee CH, Cheng HW, Gilks CB, Wang Y, Montgomery KD, Varma S, Li R, Marinelli RJ, West RB, Nielsen T, Beck AH and van de Rijn M. CSF1 expression in nongynecological leiomyosarcoma is associated with increased tumor angiogenesis. Am J Pathol. 2011; 179(4):2100-2107.

47. Ning BF, Ding J, Yin C, Zhong W, Wu K, Zeng X, Yang W, Chen YX, Zhang JP, Zhang X, Wang HY and Xie WF. Hepatocyte nuclear factor 4 alpha suppresses the development of hepatocellular carcinoma. Cancer Res. 2010; 70(19):7640-7651.
48. Kanetsky PA, Mitra N, Vardhanabhuti S, Li M, Vaughn DJ, Letrero R, Ciosek SL, Doody DR, Smith LM, Weaver J, Albano A, Chen C, Starr JR, Rader DJ, Godwin AK, Reilly MP, et al. Common variation in KITLG and at 5q31.3 predisposes to testicular germ cell cancer. Nat Genet. 2009; 41(7):811-815.
49. Huang C, Liu LY, Li ZF, Wang P, Ni L, Song LP, Xu DH and Song TS. Effects of small interfering RNAs targeting MAPK1 on gene expression profile in HeLa cells as revealed by microarray analysis. Cell Biol Int. 2008; 32(9):1081-1090.
50. Ren S, Cai HR, Li M and Furth PA. Loss of Stat5a delays mammary cancer progression in a mouse model. Oncogene. 2002; 21(27):4335-4339.
51. Xi S, Zhang Q, Gooding WE, Smithgall TE and Grandis JR. Constitutive activation of Stat5b contributes to carcinogenesis in vivo. Cancer Res. 2003; 63(20):6763-6771.
52. Nguyen DH, Martinez-Ruiz H and Barcellos-Hoff MH. Consequences of epithelial or stromal TGFbeta1 depletion in the mammary gland. J Mammary Gland Biol Neoplasia. 2011; 16(2):147-155.
53. Stoelcker B, Ruhland B, Hehlgans T, Bluethmann H, Luther T and Mannel DN. Tumor necrosis factor induces tumor necrosis via tumor necrosis factor receptor type 1-expressing endothelial cells of the tumor vasculature. Am J Pathol. 2000; 156(4):1171-1176.
54. Chu CY, Chang CC, Prakash E and Kuo ML. Connective tissue growth factor (CTGF) and cancer progression. J Biomed Sci. 2008; 15(6):675-685.
55. Kohno M and Pouyssegur J. Targeting the ERK signaling pathway in cancer therapy. Ann Med. 2006; 38(3):200-211.
56. Dentelli P, Rosso A, Olgasi C, Camussi G and Brizzi MF. IL-3 is a novel target to interfere with tumor vasculature. Oncogene. 2011; 30(50):4930-4940.
57. Klune JR, Dhupar R, Cardinal J, Billiar TR and Tsung A. HMGB1: endogenous danger signaling. Mol Med. 2008; 14(7-8):476-484.
58. Davis IJ, Kim JJ, Ozsolak F, Widlund HR, Rozenblatt-Rosen O, Granter SR, Du J, Fletcher JA, Denny CT, Lessnick SL, Linehan WM, Kung AL and Fisher DE. Oncogenic MITF dysregulation in clear cell sarcoma: defining the MiT family of human cancers. Cancer Cell. 2006; 9(6):473-484.
59. Cheng J, Ye H, Liu Z, Xu C, Zhang Z, Liu Y and Sun Y. Platelet-derived growth factor-BB accelerates prostate cancer growth by promoting the proliferation of mesenchymal stem cells. J Cell Biochem. 2013.
60. Mocellin S and Nitti D. TNF and cancer: the two sides of the coin. Front Biosci. 2008; 13:2774-2783.
61. Zong Y, Xin L, Goldstein AS, Lawson DA, Teitell MA and Witte ON. ETS family transcription factors collaborate with alternative signaling pathways to induce carcinoma from adult murine prostate cells. Proc Natl Acad Sci U S A. 2009; 106(30):12465-12470.
62. Cuevas R, Korzeniewski N, Tolstov Y, Hohenfellner M and Duensing S. FGF-2 disrupts mitotic stability in prostate cancer through the intracellular trafficking protein CEP57. Cancer Res. 2012.
63. Wang B, Xiong Q, Shi Q, Le X, Abbruzzese JL and Xie K. Intact nitric oxide synthase II gene is required for interferon-beta-mediated suppression of growth and metastasis of pancreatic adenocarcinoma. Cancer Res. 2001; 61(1):71-75.
64. Bouker KB, Skaar TC, Riggins RB, Harburger DS, Fernandez DR, Zwart A, Wang A and Clarke R. Interferon regulatory factor-1 (IRF-1) exhibits tumor suppressor activities in breast cancer associated with caspase activation and induction of apoptosis. Carcinogenesis. 2005; 26(9):1527-1535.
65. Romieu-Mourez R, Solis M, Nardin A, Goubau D, Baron-Bodo V, Lin R, Massie B, Salcedo M and Hiscott J. Distinct roles for IFN regulatory factor (IRF)-3 and IRF-7 in the activation of antitumor properties of human macrophages. Cancer Res. 2006; 66(21):10576-10585.
66. Hu X, Yang D, Zimmerman M, Liu F, Yang J, Kannan S, Burchert A, Szulc Z, Bielawska A, Ozato K, Bhalla K and Liu K. IRF8 regulates acid ceramidase expression to mediate apoptosis and suppresses myelogeneous leukemia. Cancer Res. 2011; 71(8):2882-2891.
67. Hayami S, Yoshimatsu M, Veerakumarasivam A, Unoki M, Iwai Y, Tsunoda T, Field HI, Kelly JD, Neal DE, Yamaue H, Ponder BA, Nakamura Y and Hamamoto R. Overexpression of the JmjC histone demethylase KDM5B in human carcinogenesis: involvement in the proliferation of cancer cells through the E2F/RB pathway. Mol Cancer. 2010; 9:59.
68. Bradham C and McClay DR. p38 MAPK in development and cancer. Cell Cycle. 2006; 5(8):824-828.

69. Bhalla K, Hwang BJ, Dewi RE, Ou L, Twaddel W, Fang HB, Vafai SB, Vazquez F, Puigserver P, Boros L and Girnun GD. PGC1alpha promotes tumor growth by inducing gene expression programs supporting lipogenesis. Cancer Res. 2011; 71(21):6888-6898.
70. Zhang J, Li H, Yu JP, Wang SE and Ren XB. Role of SOCS1 in tumor progression and therapeutic application. Int J Cancer. 2012; 130(9):1971-1980.
71. Hix LM, Karavitis J, Khan MW, Shi YH, Khazaie K and Zhang M. Tumor STAT1 Transcription Factor Activity Enhances Breast Tumor Growth and Immune Suppression Mediated by Myeloid-derived Suppressor Cells. J Biol Chem. 2013; 288(17):11676-11688.
72. Rakoff-Nahoum S and Medzhitov R. Toll-like receptors and cancer. Nat Rev Cancer. 2009; 9(1):57-63.
73. Oblak A and Jerala R. Toll-like receptor 4 activation in cancer progression and therapy. Clin Dev Immunol. 2011; 2011:609579.
74. Vacas E, Bajo AM, Schally AV, Sanchez-Chapado M, Prieto JC and Carmena MJ. Vasoactive intestinal peptide induces oxidative stress and suppresses metastatic potential in human clear cell renal cell carcinoma. Mol Cell Endocrinol. 2013; 365(2):212-222.
75. Gilligan TD, Hayes DF, Seidenfeld J and Temin S. ASCO Clinical Practice Guideline on Uses of Serum Tumor Markers in Adult Males With Germ Cell Tumors. J Oncol Pract. 2010; 6(4):199-202.
76. Zheng LD, Yang CL, Qi T, Qi M, Tong L and Tong QS. Effects of resistin-like molecule beta over-expression on gastric cancer cells in vitro. World J Gastroenterol. 2012; 18(8):754-766.
77. Cook WD, McCaw BJ, Herring C, John DL, Foote SJ, Nutt SL and Adams JM. PU.1 is a suppressor of myeloid leukemia, inactivated in mice by gene deletion and mutation of its DNA binding domain. Blood. 2004; 104(12):3437-3444.
78. de Leval L, Rickman DS, Thielen C, Reynies A, Huang YL, Delsol G, Lamant L, Leroy K, Briere J, Molina T, Berger F, Gisselbrecht C, Xerri L and Gaulard P. The gene expression profile of nodal peripheral T-cell lymphoma demonstrates a molecular link between angioimmunoblastic T-cell lymphoma (AITL) and follicular helper T (TFH) cells. Blood. 2007; 109(11):4952-4963.
79. Wood KJ, Feng G, Wei B, Sawitzki B and Bushell AR. Interferon gamma: friend or foe? Transplantation. 2007; 84(1 Suppl):S4-5.
80. Nakaji M, Yano Y, Ninomiya T, Seo Y, Hamano K, Yoon S, Kasuga M, Teramoto T, Hayashi Y and Yokozaki H. IFN-alpha prevents the growth of pre-neoplastic lesions and inhibits the development of hepatocellular carcinoma in the rat. Carcinogenesis. 2004; 25(3):389-397.

Supplemental Table 7. Twelve key genes involved in age-dependent spleen changes in the presence of a tumor. These 12 genes were determined by finding the common genes for the spleen of LLC tumor bearing C57BL/6 old male mice (O) versus all other ages (Adolescent (A), Young Adult (Y), and Middle-Aged (M)) that are in common between the significant upstream regulators and the biofunction analysis. The second column denotes the effects these genes have on tumor progression based on the literature. The genes in bold and italic are found to overlap with genes found in the *Gene Set Enrichment Analysis (GSEA) for GO gene sets with a FWER < 0.05 for old tumor samples vs other aged spleen samples. Genes with no reported effects on tumors were identified as Not Determined (ND).*

Gene Name	Effects on Tumor (Ref.)	Log_2 Fold Change					
		Y vs A	M vs A	M vs YA	O vs A	O vs Y	O vs M
APOE	Inhibits [1]	-0.0354	0.2486	0.2840	0.8393	0.8747	0.5907
C4A/C4B	Promotes [2]	-0.2148	0.1296	0.3444	0.8243	1.0391	0.6947
CCL19	Inhibits [3]	-0.1713	-0.0742	0.0970	0.9392	1.1105	1.0135
CCL5	Promotes [4]	-0.0459	0.7019	0.7478	1.7339	1.7798	1.0320
CCND1	Promotes [5]	-0.0018	0.2661	0.2679	0.6441	0.6459	0.3780
CD2	Inhibits [6]	0.0257	0.1913	0.1656	0.9362	0.9105	0.7449
CD3E	Inhibits [7]	-0.0755	0.1223	0.1978	0.8525	0.9280	0.7302
CD72	ND	-0.1454	0.3245	0.4699	1.0844	1.2299	0.7600
CSK	Inhibits [8]	-0.0163	0.0720	0.0883	0.6276	0.6439	0.5556
CXCR3	Promotes [9]	0.0367	0.1473	0.1106	0.7730	0.7363	0.6257
SFRP1	Inhibits [10]	-0.0970	0.0288	0.1258	0.4718	0.5688	0.4430
TRAF1	Inhibits [11]	-0.0198	0.2710	0.2908	0.5947	0.6145	0.3237

1. Pola C. Cancer: Antitumor duality of ApoE. Nat Med. 2012; 18(12):1752.
2. Zafar GI, Grimm EA, Wei W, Johnson MM and Ellerhorst JA. Genetic deficiency of complement isoforms C4A or C4B predicts improved survival of metastatic renal cell carcinoma. J Urol. 2009; 181(3):1028-1034; discussion 1034.
3. Hillinger S, Yang SC, Batra RK, Strieter RM, Weder W, Dubinett SM and Sharma S. CCL19 reduces tumour burden in a model of advanced lung cancer. Br J Cancer. 2006; 94(7):1029-1034.
4. Huang CY, Fong YC, Lee CY, Chen MY, Tsai HC, Hsu HC and Tang CH. CCL5 increases lung cancer migration via PI3K, Akt and NF-kappaB pathways. Biochem Pharmacol. 2009; 77(5):794-803.
5. Roy PG, Pratt N, Purdie CA, Baker L, Ashfield A, Quinlan P and Thompson AM. High CCND1 amplification identifies a group of poor prognosis women with estrogen receptor positive breast cancer. Int J Cancer. 2010; 127(2):355-360.
6. Sarkar S, Ghosh A, Mukherjee J, Chaudhuri S and Chaudhuri S. CD2-SLFA3/T11TS interaction facilitates immune activation and glioma regression by apoptosis. Cancer Biol Ther. 2004; 3(11):1121-1128.
7. Huang Y, Lin L, Shanker A, Malhotra A, Yang L, Dikov MM and Carbone DP. Resuscitating cancer immunosurveillance: selective stimulation of DLL1-Notch signaling in T cells rescues T-cell function and inhibits tumor growth. Cancer Res. 2011; 71(19):6122-6131.
8. Nakagawa T, Tanaka S, Suzuki H, Takayanagi H, Miyazaki T, Nakamura K and Tsuruo T. Overexpression of the csk gene suppresses tumor metastasis in vivo. Int J Cancer. 2000; 88(3):384-391.
9. Murakami T, Kawada K, Iwamoto M, Akagami M, Hida K, Nakanishi Y, Kanda K, Kawada M, Seno H, Taketo MM and Sakai Y. The role of CXCR3 and CXCR4 in colorectal cancer metastasis. Int J Cancer. 2013; 132(2):276-287.
10. Klopocki E, Kristiansen G, Wild PJ, Klaman I, Castanos-Velez E, Singer G, Stohr R, Simon R, Sauter G, Leibiger H, Essers L, Weber B, Hermann K, Rosenthal A, Hartmann A and Dahl E. Loss of SFRP1 is associated with breast cancer progression and poor prognosis in early stage tumors. Int J Oncol. 2004; 25(3):641-649.

11. Rajandram R, Bennett NC, Wang Z, Perry-Keene J, Vesey DA, Johnson DW and Gobe GC. Patient samples of renal cell carcinoma show reduced expression of TRAF1 compared with normal kidney and functional studies in vitro indicate TRAF1 promotes apoptosis: potential for targeted therapy. Pathology. 2012; 44(5):453-459.

Supplemental Table 8. Key genes involved in age-dependent spleen changes in non-tumor bearing male C57BL/6 mice. These key genes were determined by finding the common genes for old spleen samples (O) versus all other ages (Adolescent (A), Young Adult (Y), and Middle-Aged (M)) that are in common between the significant upstream regulators and the biofunction analysis. The second column denotes the effects these genes have on tumor progression based on the literature. The genes in bold and italic are found to overlap with genes found in the Gene Set Enrichment Analysis (GSEA) for GO gene sets with a FDR < 0.05 for old tumor samples vs other aged spleen samples.

**Gene Name**	**Effects on Tumor (Ref.)**	**Log_2 Fold Change**					
		**O vs M**	**O vs Y**	**O vs A**	**M vs Y**	**M vs A**	**Y vs A**
AXL	Promotes [1]	0.313	0.682	0.947	0.369	0.634	0.265
BAX	Inhibits [2]	-0.607	-0.263	-1.322	0.344	-0.715	-1.059
C1QA	Promotes [3]	0.760	0.731	0.809	-0.030	0.049	0.079
CCL5	Promotes [4]	0.983	0.906	0.818	-0.078	-0.165	-0.087
CHST1	Promotes [5]	0.511	0.589	0.868	0.078	0.357	0.279
CLEC2D	Promotes [6]	-0.950	-0.788	-2.170	0.162	-1.220	-1.382
EFNB1	Promotes [7]	0.701	0.439	0.507	-0.262	-0.194	0.067
HOXA5	Inhibits [8]	0.275	0.497	0.618	0.221	0.343	0.122
HOXB7	Promotes [9]	0.281	0.397	0.469	0.116	0.188	0.073
LGALS1	Promotes [10]	1.073	0.747	0.561	-0.326	-0.512	-0.186
LGALS3	Promotes [11]	0.770	0.305	0.688	-0.465	-0.082	0.384
NFE2L1	Inhibits [12]	0.392	0.361	0.345	-0.031	-0.046	-0.015
NKX2-3	Inhibits [13]	0.346	0.319	0.319	-0.028	-0.028	0.000
NT5E	Promotes [14]	-0.370	0.408	0.610	0.778	0.980	0.201
S100A6	Promotes [15]	1.257	0.660	0.810	-0.596	-0.446	0.150
SFRP1	Inhibits [16]	0.398	0.533	0.846	0.135	0.448	0.313
SLC1A3	Promotes [17]	0.656	0.582	0.871	-0.073	0.215	0.289
TCF21	Inhibits [18]	0.869	0.747	0.993	-0.122	0.124	0.245
TGM2	Promotes [19]	0.616	0.520	0.883	-0.097	0.267	0.364
XBP1	Promotes [20]	-0.305	0.452	0.942	0.757	1.247	0.490
YBX3	Promotes [21]	-0.933	-0.394	-0.728	0.539	0.206	-0.333

1. Li Y, Ye X, Tan C, Hongo JA, Zha J, Liu J, Kallop D, Ludlam MJ and Pei L. Axl as a potential therapeutic target in cancer: role of Axl in tumor growth, metastasis and angiogenesis. Oncogene. 2009; 28(39):3442-3455.
2. Degenhardt K, Chen G, Lindsten T and White E. BAX and BAK mediate p53-independent suppression of tumorigenesis. Cancer Cell. 2002; 2(3):193-203.
3. Teschendorff AE and Caldas C. A robust classifier of high predictive value to identify good prognosis patients in ER-negative breast cancer. Breast Cancer Res. 2008; 10(4):R73.
4. Huang CY, Fong YC, Lee CY, Chen MY, Tsai HC, Hsu HC and Tang CH. CCL5 increases lung cancer migration via PI3K, Akt and NF-kappaB pathways. Biochem Pharmacol. 2009; 77(5):794-803.
5. Li X, Tu L, Murphy PG, Kadono T, Steeber DA and Tedder TF. CHST1 and CHST2 sulfotransferase expression by vascular endothelial cells regulates shear-resistant leukocyte rolling via L-selectin. J Leukoc Biol. 2001; 69(4):565-574.
6. Coulouarn C, Factor VM, Conner EA and Thorgeirsson SS. Genomic modeling of tumor onset and progression in a mouse model of aggressive human liver cancer. Carcinogenesis. 2011; 32(10):1434-1440.
7. Kataoka H, Tanaka M, Kanamori M, Yoshii S, Ihara M, Wang YJ, Song JP, Li ZY, Arai H, Otsuki Y, Kobayashi T, Konno H, Hanai H and Sugimura H. Expression profile of EFNB1, EFNB2, two ligands of EPHB2 in human gastric cancer. J Cancer Res Clin Oncol. 2002; 128(7):343-348.
8. Chen H, Chung S and Sukumar S. HOXA5-induced apoptosis in breast cancer cells is mediated by caspases 2 and 8. Mol Cell Biol. 2004; 24(2):924-935.

9. Jin K, Kong X, Shah T, Penet MF, Wildes F, Sgroi DC, Ma XJ, Huang Y, Kallioniemi A, Landberg G, Bieche I, Wu X, Lobie PE, Davidson NE, Bhujwalla ZM, Zhu T, et al. The HOXB7 protein renders breast cancer cells resistant to tamoxifen through activation of the EGFR pathway. Proc Natl Acad Sci U S A. 2012; 109(8):2736-2741.
10. Hsu YL, Wu CY, Hung JY, Lin YS, Huang MS and Kuo PL. Galectin-1 promotes lung cancer tumor metastasis by potentiating integrin alpha6beta4 and Notch1/Jagged2 signaling pathway. Carcinogenesis. 2013; 34(6):1370-1381.
11. Cheng CL, Hou HA, Lee MC, Liu CY, Jhuang JY, Lai YJ, Lin CW, Chen HY, Liu FT, Chou WC, Chen CY, Tang JL, Yao M, Huang SY, Ko BS, Wu SJ, et al. Higher bone marrow LGALS3 expression is an independent unfavorable prognostic factor for overall survival in patients with acute myeloid leukemia. Blood. 2013; 121(16):3172-3180.
12. Oh DH, Rigas D, Cho A and Chan JY. Deficiency in the nuclear-related factor erythroid 2 transcription factor (Nrf1) leads to genetic instability. FEBS J. 2012; 279(22):4121-4130.
13. Wang X, Zbou C, Qiu G, Fan J, Tang H and Peng Z. Screening of new tumor suppressor genes in sporadic colorectal cancer patients. Hepatogastroenterology. 2008; 55(88):2039-2044.
14. Wang H, Lee S, Nigro CL, Lattanzio L, Merlano M, Monteverde M, Matin R, Purdie K, Mladkova N, Bergamaschi D, Harwood C, Syed N, Szlosarek P, Briasoulis E, McHugh A, Thompson A, et al. NT5E (CD73) is epigenetically regulated in malignant melanoma and associated with metastatic site specificity. Br J Cancer. 2012; 106(8):1446-1452.
15. Ning X, Sun S, Zhang K, Liang J, Chuai Y, Li Y and Wang X. S100A6 protein negatively regulates CacyBP/SIP-mediated inhibition of gastric cancer cell proliferation and tumorigenesis. PLoS One. 2012; 7(1):e30185.
16. Klopocki E, Kristiansen G, Wild PJ, Klaman I, Castanos-Velez E, Singer G, Stohr R, Simon R, Sauter G, Leibiger H, Essers L, Weber B, Hermann K, Rosenthal A, Hartmann A and Dahl E. Loss of SFRP1 is associated with breast cancer progression and poor prognosis in early stage tumors. Int J Oncol. 2004; 25(3):641-649.
17. Pavlides S, Tsirigos A, Vera I, Flomenberg N, Frank PG, Casimiro MC, Wang C, Pestell RG, Martinez-Outschoorn UE, Howell A, Sotgia F and Lisanti MP. Transcriptional evidence for the "Reverse Warburg Effect" in human breast cancer tumor stroma and metastasis: similarities with oxidative stress, inflammation, Alzheimer's disease, and "Neuron-Glia Metabolic Coupling". Aging (Albany NY). 2010; 2(4):185-199.
18. Ye YW, Jiang ZM, Li WH, Li ZS, Han YH, Sun L, Wang Y, Xie J, Liu YC, Zhao J, Tang AF, Li XX, Guan ZC, Gui YT and Cai ZM. Down-regulation of TCF21 is associated with poor survival in clear cell renal cell carcinoma. Neoplasma. 2012; 59(6):599-605.
19. Miyoshi N, Ishii H, Mimori K, Tanaka F, Hitora T, Tei M, Sekimoto M, Doki Y and Mori M. TGM2 is a novel marker for prognosis and therapeutic target in colorectal cancer. Ann Surg Oncol. 2010; 17(4):967-972.
20. Romero-Ramirez L, Cao H, Nelson D, Hammond E, Lee AH, Yoshida H, Mori K, Glimcher LH, Denko NC, Giaccia AJ, Le QT and Koong AC. XBP1 is essential for survival under hypoxic conditions and is required for tumor growth. Cancer Res. 2004; 64(17):5943-5947.
21. Wang GR, Zheng Y, Che XM, Wang XY, Zhao JH, Wu KJ, Zeng J, Pan CE and He DL. Upregulation of human DNA binding protein A (dbpA) in gastric cancer cells. Acta Pharmacol Sin. 2009; 30(10):1436-1442.
